# “*Inside CKD*” Multinational-Microsimulation Modelling Insights Into the Increasing CKD Burden

**DOI:** 10.1016/j.ekir.2025.07.014

**Published:** 2025-07-18

**Authors:** Jay Wish, Saeed MG Al-Ghamdi, Jean-Michel Halimi, Michel Jadoul, Vivekanand Jha, You-Seon Nam, Juan F Navarro-González, Lise Retat, Laura Webber, Claudia Cabrera

**Affiliations:** 1 Department of Medicine, School of Medicine, Indiana University, Indianapolis, Indiana, USA; 2 Department of Medicine, King Faisal Specialist Hospital and Research Centre, Jeddah, Saudi Arabia; 3 Service de Néphrologie-Immunologie Clinique, Hôpital Bretonneau, CHRU Tours, Tours, France; 4 Department of Nephrology, Cliniques universitaires Saint-Luc, Université Catholique de Louvain, Brussels, Belgium; 5 Faculty of Medicine, School of Public Health, Imperial College London, London, UK; 6 Manipal Academy of Higher Education, Manipal, Karnataka, India; 7 Global Medical Affairs, BioPharmaceuticals Medical, AstraZeneca, Gaithersburg, Maryland, USA; 8 Unidad de Investigación y Servicio de Nefrología, Hospital Universitario Nuestra Señora de Candelaria, Santa Cruz de Tenerife, Islas Canarias, Spain; 9 RICORS2040 (RD24/0004/0022), Instituto de Salud Carlos III, Madrid, Spain; 10 Instituto de Tecnologías Biomédicas, Universidad de La Laguna, San Cristóbal de La Laguna, Tenerife, Spain; 11 Facultad de Ciencias de la Salud, Universidad Fernando Pessoa Canarias, Las Palmas de Gran Canaria, Spain; 12 HealthLumen Ltd, London, UK; 13 Real World Science and Analytics, BioPharmaceuticals Medical, AstraZeneca, Gothenburg, Sweden

**Keywords:** burden of disease, chronic kidney disease (CKD), health care systems, *Inside CKD*, microsimulation modeling, screening

## Abstract

**Introduction:**

Chronic kidney disease (CKD) affects approximately 1 in 10 people worldwide and is associated with increased morbidity and mortality, yet it remains underdiagnosed, undertreated, and underrecognized compared with other noncommunicable diseases.

**Methods:**

The *Inside CKD* program uses a microsimulation model to project the clinical and economic burden of CKD. Here, we assessed published *Inside CKD* data*,* from 31 countries and regions, in the context of existing guidelines, recommendations, and other global initiatives.

**Results:**

Our findings highlight that, if current low rates of diagnosis are maintained, the CKD population is projected to increase by 5.8%, with associated health care costs increasing by 9.3%, by 2027. Screening for CKD is also projected to be cost-effective and to modestly increase life expectancy.

**Conclusion:**

Implementing screening and treatment strategies will be crucial for the early identification of CKD and for overcoming the current clinical inertia around the diagnosis and management of CKD.

## Graphical abstract

**Figure:**
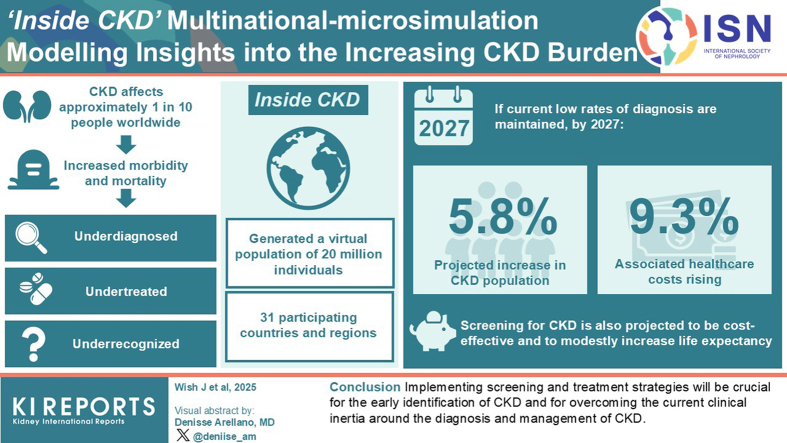

See Commentary on Page 3297

### CKD: Where are we Now?

CKD is a global public health concern that affects approximately 1 in 10 people worldwide.^1^, ^2^, ^3^, ^4^, ^5^ It is defined by abnormalities of kidney function or structure that are present for > 3 months and that have implications for health.^6^ CKD is a progressive condition, and disease severity is stratified based on estimated glomerular filtration rate (eGFR) and albuminuria measurements.^6^ CKD incurs substantial morbidity and mortality, with related deaths increasing 2- to 3-fold over the past 30 years,^1^^,^^5^ making it one of the few noncommunicable diseases with rising mortality.^7^ Indeed, CKD is projected to increase from the 16th leading cause of death in 2016 to the fifth leading cause by 2040.^8^ Furthermore, the global population is aging, with 1 in 5 people predicted to be aged ≥ 60 years by 2050^9^; a population predisposed to CKD.^5^^,^^10^ Despite this, CKD receives less attention and fewer resources than other noncommunicable diseases.^11^ Low awareness of CKD among health care professionals, policymakers, and patients is reported to contribute to its lower profile and research prioritization,^12^ even though advanced CKD treatment constitutes approximately 2% to 5% of health care budgets worldwide.^13^ Finally, although CKD increases the risk of severe COVID-19,^14^ early reports neglected the impact of kidney disease versus diabetes or autoimmune conditions on COVID-19 infection.^15^^,^^16^

Sociopolitical factors, including the COVID-19 pandemic and geopolitical tensions, have compromised global health care budgets,^17^ meaning that policies to improve CKD outcomes need to be developed and implemented amidst multiple competing priorities. Recently, an international consensus from nephrology societies urged for CKD to be prioritized on the global health agenda and to be recognized as a major driver of premature death by the World Health Organization.^18^ Furthermore, the European Kidney Health Alliance released a manifesto advocating a united strategy to prioritize kidney care.^19^ Although high-income nations can reallocate resources to support CKD initiatives, patients from disadvantaged groups may experience disparities in CKD care and outcomes.^20^, ^21^, ^22^ Low- and middle-income nations may not be able to implement international recommendations for CKD because of deep-rooted and pervasive health inequities.^23^^,^^24^ For example, kidney replacement therapy (KRT) is required for kidney failure; however, individuals must assume financial responsibility for this treatment when health care systems are unable to fund KRT.^25^ Countries without the resources to treat late-stage CKD usually have lower wages (median: < US$2000/yr), and so this creates severe financial hardship given the relatively high cost of dialysis.^3^^,^^26^ Kidney failure can equate to death in these circumstances, with reports that approximately 96% of patients in sub-Saharan Africa who could not access dialysis died or were presumed dead.^27^

Responsive health care policies to anticipate and navigate trends, even with limited country-specific data, can play a pivotal role in CKD management. Microsimulation models can complement real-world studies by creating virtual populations derived from existing data, the trajectories for which are modelled for risk factors of given diseases over projected time periods to predict future health outcomes and costs.^28^^,^^29^ Although many CKD models are available, few include all necessary variables for accurate predictions. A systematic review of 13 models revealed that many did not include both albuminuria and eGFR measurements, even though both factors are required for accurate CKD stratification, and that these models did not adequately address patient heterogeneity and disease aetiology.^29^

### Introducing *Inside CKD*

*Inside CKD* is a novel program that uses a microsimulation approach to address these limitations and to project the burden of CKD. It can also explore the potential impact of hypothetical interventions, such as screening, on health outcomes. The model is built on a validated population-based microsimulation.^30^, ^31^, ^32^, ^33^, ^34^, ^35^, ^36^ The methodology (Figure 1),^37^, ^38^, ^39^ validation, and sensitivity analyses of *Inside CKD* have been previously described.^36^, ^37^, ^38^ In brief, *Inside CKD* generated a virtual population of 20 million individuals for each of the 31 participating countries and regions. Each hypothetical person was assigned key demographic characteristics, a CKD health status and comorbidities, and was cycled through the microsimulation in 1-year increments. The time horizon could be adapted depending on the analysis; 2022 to 2027 was used for the burden of disease outcomes,^37^^,^^38^^,^^40^ whereas the intervention module was assessed over a lifetime horizon (from first intervention to death of each individual).^39^

**Figure 1:**
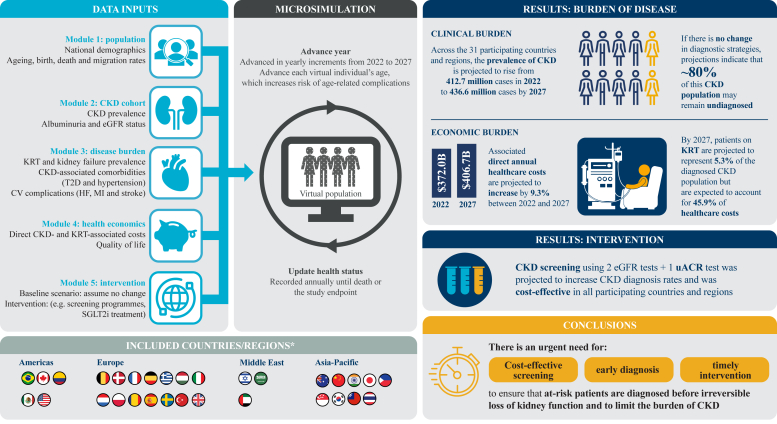
Infographic illustrating the methodology of the *Inside CKD* microsimulation model. ∗Americas [left to right]: Brazil, Canada, Colombia, Mexico, USA; Europe [left to right]: Belgium, Denmark, France, Germany, Greece, Hungary, Italy, Netherlands, Poland, Romania, Spain, Sweden, Türkiye, UK; Middle East [left to right]: Israel, Saudi Arabia, United Arab Emirates; Asia-Pacific [left to right]: Australia, China, India, Japan, Philippines, Singapore, South Korea, Taiwan, Thailand. CKD, chronic kidney disease; CV, cardiovascular; eGFR, estimated glomerular filtration rate; HF, heart failure; KRT, kidney replacement therapy; MI, myocardial infarction; SGLT2i, sodium-glucose cotransporter 2 inhibitor; T2D, type 2 diabetes; uACR, urine albumin–creatinine ratio. The data presented in this figure have been taken from Chertow *et al.*^37^ (Figure 2, Table 3), Chadban *et al.*^38^ (Figure 1, Figure 2, Supplementary Tables S3 and S6), and Tangri *et al.*^39^ (Figure 3). All of these works are under a CC-BY license http://creativecommons.org/licenses/by/4.0/.

A literature search was undertaken to assign relevant prospective demographic and disease prevalence data for each virtual population.^36^^,^^41^ Baseline demographics were derived from national statistics for each country/region (Figure 1). Albuminuria and eGFR were also country-specific or were calculated from aggregated national CKD prevalence data if no information was available. Each individual was assigned a CKD status based on their eGFR or albuminuria status (i.e., CKD stages 1–5 or no CKD); the cohort with CKD was stratified according to Kidney Disease: Improving Global Outcomes (KDIGO) guidelines.^6^^,^^42^ New KDIGO guidelines have been published since the *Inside CKD* microsimulations were conducted; however, the CKD staging criteria remain the same.^6^ The CKD cohort was stratified into diagnosed and undiagnosed cases using national diagnosis rates, when available. The frequency of key comorbidities, cardiovascular complications, and all-cause mortality (adjusted for age and sex [biological sex]) were based on national data if known (Figure 1). Proxy data inputs were used if no country-specific data were available.

A multidimensional algorithm was used to identify potential proxy data, which were then confirmed as suitable by an expert steering committee.^36^ A paucity of available input data is a limitation of any model; however, the *Inside CKD* model can be updated as new data become available, and the literature search highlights knowledge gaps for further research.

Virtual individuals progressed through the microsimulation in yearly increments. Annually, each individual’s health status changed according to regression coefficients of eGFR trajectories that were based on their clinical characteristics, such as age, sex, and comorbidities. CKD progression was modelled using eGFR slopes based on data from the published DISCOVER CKD database,^36^^,^^43^ and adapted to account for clinical characteristics. Model outputs included the prevalence and incidence for each disease state, comorbidity, complication, and/or health outcome.

*Inside CKD* did not factor pharmaceutical management into the burden of disease analysis. However, for the intervention module, treatment with a renin-angiotensin system inhibitor (RASi) was assumed according to national eligibility criteria. Hemodialysis, peritoneal dialysis, or kidney transplantation (including maintenance) were assumed to be the 3 treatment options for kidney failure. The analysis adopted a national health care system perspective for most countries, a commercial setting for the USA, and a mixed private–public framework for India. Given the multifaceted variability between countries, direct comparisons should be made with caution. Cost analyses were informed by a cost library,^41^ which identified per-patient treatment costs for CKD stages 3a to 5 (CKD stages 1 and 2 were deemed cost-neutral, so projected outputs were conservative), KRT, and cardiovascular complications. These costs were standardized from local currencies to international dollars, a hypothetical currency that has the same purchasing power parity as the US dollar at a specific time (2022 was used for *Inside CKD*). Hospitalization costs were excluded because of the risk of confounding with other conditions or complications, and indirect costs were excluded because of insufficient data.

This review summarizes findings from *Inside CKD* in the context of existing guidelines, recommendations, and other global initiatives to identify knowledge gaps, highlight unmet needs, and inform evidence-based policies.

## Methods

### Search Strategy and Selection Criteria

The first aim of the search strategy was to retrieve all published data from the *Inside CKD* program. Citations were identified through a search of the PubMed database conducted on 31 January 2025 using the terms “*Inside CKD*” and “microsimulation AND Chronic Kidney Disease OR CKD” without limitations. Only full publications were considered. The authors of this review, who were members of the *Inside CKD* Scientific Steering Committee, reviewed and approved the final list of *Inside CKD* publications.

A second aim was to identify relevant guidelines, recommendations, as well as economic and epidemiological data to contextualize the *Inside CKD* research. Medline-indexed publications were retrieved using a PubMed search with the search terms provided in Supplementary Table S1. English language citations from January 1, 2013 to January 31, 2025 were retrieved. We also used the KDIGO guidelines to cross-check for earlier pivotal publications.^6^^,^^42^ An additional search focused on relevant grey literature; the authors discussed and agreed on a list of key websites (Supplementary Table S2), which were manually searched for relevant policy materials and/or global burden of disease data. Finally, the authors contributed references based on their own expertise.

All publications retrieved using the above strategy were exported to a Microsoft Excel file and screened for consideration by 2 independent reviewers. Any variance was resolved by the lead author (JW). The inclusion criteria comprised publications that provided global epidemiological data, international management guidelines, commentaries, or global recommendations for managing CKD. Local guidelines and policies were not considered to be within scope unless deemed to have significant international influence or to provide novel insights for ≥ 1 of the 31 *Inside CKD* countries and regions. The full inclusion criteria are provided in Supplementary Table S3. A summary of the publications identified, screened, and included in this review is shown in Supplementary Figure S1.

### Role of the Funding Source

Members of the funding source contributed to the methods, interpretation, and writing of the paper and funded further editorial support. CC and YSN were employed by the funding source at the time of writing. Authors had access to all materials and contributed substantively to the development of the search methodology, outline, and/or subsequent drafts. The corresponding author had final responsibility for the decision to submit for publication.

## Results And Discussion

### Results of Literature Search for Inside CKD

We identified 6 publications from the *Inside CKD* program for our review. These publications included a methodological paper,^36^ a review of CKD medical costs across the 31 participating countries and regions,^41^ 2 complementary papers reporting clinical and economic projections,^37^^,^^38^ a paper exploring the impact of urine albumin–creatinine ratio (uACR) categories on CKD-related outcomes,^40^ and a cost-effectiveness analysis of 2 screening strategies.^39^

### What is the Future of CKD if Nothing Changes?

**Figure 2:**
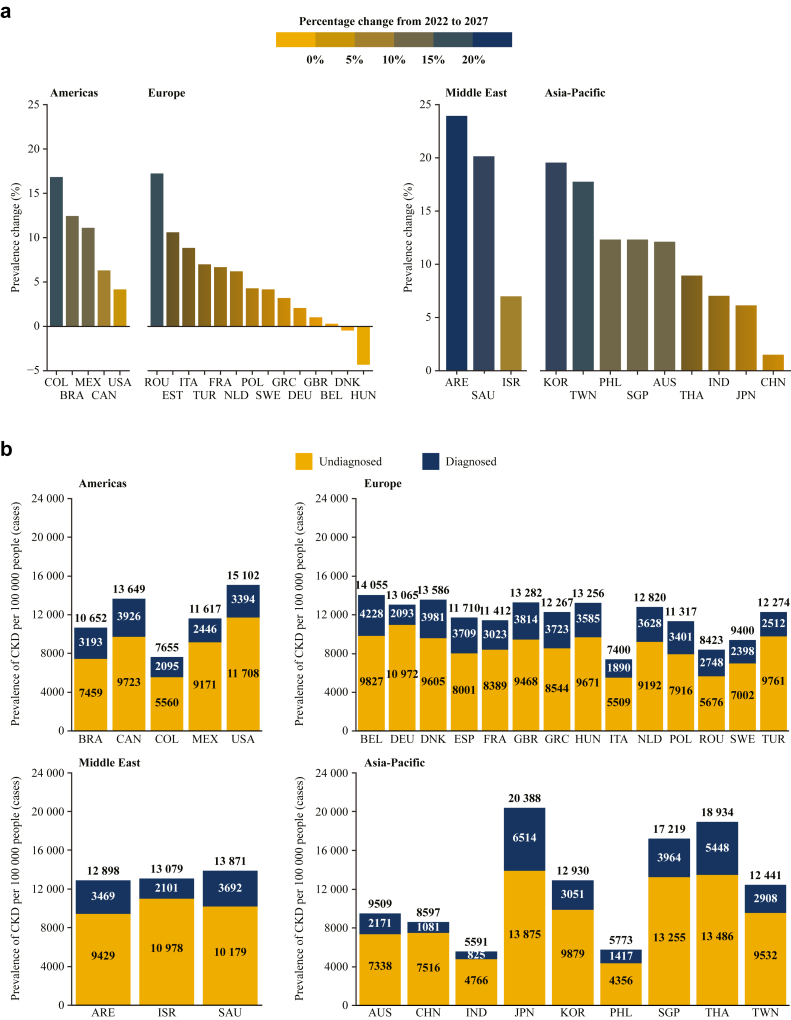
(a) Projected percentage change of CKD prevalence from 2022 to 2027 (b) projected prevalence of diagnosed and undiagnosed CKD rates per 100,000 people in 2027. ARE, United Arab Emirates; AUS, Australia; BEL, Belgium; BRA, Brazil; CAN, Canada; CHN, China; CKD, chronic kidney disease; COL, Colombia; DEU, Germany; DNK, Denmark; ESP, Spain; FRA, France; GBR, United Kingdom; GRC, Greece; HUN, Hungary; IND, India; ISR, Israel; ITA, Italy; JPN, Japan; KOR, South Korea; MEX, Mexico; NLD, Netherlands; PHL, Philippines; POL, Poland; ROU, Romania; SAU, Saudi Arabia; SGP, Singapore; SWE, Sweden; THA, Thailand; TUR, Türkiye; TWN, Taiwan; UAE, United Arab Emirates; USA, United States of America. Note: The United Arab Emirates has a large and diverse Expatriate population with a different CKD profile; only the Emirati population has been presented here. The data presented in this figure have been taken from Chertow *et al.*^37^ (Figure 2, Table 3). This work is licensed under a CC-BY license http://creativecommons.org/licenses/by/4.0/.

The future projected by *Inside CKD* is bleak if current trends continue; by 2027, most countries and regions were projected to have an increased prevalence of CKD (Figure 2a).^37^ Trends were influenced by national demographics; hence, some countries showed a slight decrease in absolute numbers of CKD cases because of overall population decline. The prevalence of known demographic drivers of CKD (e.g., the proportion of individuals aged ≥ 65 years, smoking rates, and prevalence of obesity) varied between countries, but each virtual population had a high frequency of at least 1 concerning parameter at baseline (Supplementary Table S4). Regarding disease severity, most CKD cases were moderate (CKD stage 3) (Supplementary Table S5), reflecting the current real-world situation.^2^^,^^4^

The proportion of people with undiagnosed CKD was consistently high across the *Inside CKD* cohort and projected to represent approximately 80% of the CKD population by 2027 (Figure 2b and Supplementary Table S5).^37^ Early-stage CKD is often asymptomatic. If symptoms are present, then they are often nondescript (e.g., fatigue) and can be falsely ascribed to other lifestyle factors or conditions without testing for CKD.^6^ Across the *Inside CKD* cohort, moderate-to-severe CKD (CKD stages 3a–5) was more likely to be diagnosed than mild CKD, representing a missed opportunity to slow the irreversible loss of kidney function. Although progressive decline in kidney function may be inevitable, the rate of deterioration can be influenced. Real-world data suggest that a delayed diagnosis of just 1 year for patients with CKD stage 3 increases the risk of progression to CKD stages 4 or 5 by 40%.^44^ Conversely, early intervention may delay eGFR decline.^45^

Ideally, CKD treatment should begin before the onset of substantially reduced eGFR or elevated albuminuria. In 2020, a multinational, observational cohort study found that sodium-glucose cotransporter 2 inhibitors (SGLT2is) produced substantial benefits across all eGFR categories.^46^ Similar findings have been reported for RASis, with the greatest benefits observed in the earliest stages of disease.^47^ A 2019 meta-analysis reported that a slower decline in eGFR was associated with a lower risk of kidney failure, even in patients with eGFR ≥ 60 ml/min per 1.73 m^2^.^48^ Furthermore, *post hoc* trial analyses highlight the potential delays to CKD progression and kidney failure associated with SGLT2i treatment, the implementation of which could lead to notable patient, societal, health care, and economic benefits.^49^

Progression of CKD has a substantial impact on quality of life, which is driven by the presence of comorbidities and the increased incidence of cardiovascular complications.^50^ *Inside CKD* projects that, between 2022 and 2027, there will be a high cumulative incidence of cardiovascular disease (CVD) in the diagnosed CKD population (Supplementary Figure S2).^37^ Patients with undiagnosed CKD are likely to be affected to a similar or greater extent because they will not receive the benefits of regular monitoring that facilitates CVD diagnosis and care. Projected all-cause mortality in 2022 was higher in undiagnosed than in diagnosed individuals for most countries or regions (Supplementary Figure S3),^37^ underscoring that mild-to-moderate forms of CKD (CKD stages 1–3a) warrant clinical concern. Notably, the microsimulation did not account for the COVID-19 pandemic and so may underestimate the true mortality because of CKD, given that CKD increases the risk of COVID-19-related mortality.^16^

The resources available to address the suboptimal management of mild-to-moderate CKD vary between nations. An absence of functional surveillance systems and fragmented or inadequate health care infrastructure, particularly at the primary care level, compounded by a lack of basic diagnostic testing, compromise the likelihood of early diagnosis.^51^ Only one-third of low-income countries have the means to measure serum creatinine in primary care, with no available eGFR or uACR testing.^52^ The impact of limited resources could be exacerbated by the potentially poorer health status of the general population in lower-income versus higher-income regions. For patients with kidney failure, a dearth of nephrology health care professionals and poor availability of KRT pose considerable concern.^3^^,^^18^^,^^52^^,^^53^ A survey of 167 countries found that the distribution of nephrologists varied substantially, with < 1 nephrologist per 100,000 people in many low-income countries.^3^

### Assessing the Cost of Maintaining the Current Situation

To estimate future CKD expenditure, *Inside CKD* projected the annual direct costs of diagnosed CKD and associated complications. Findings from the microsimulation cost library illustrate substantial variation between regions in per-patient spending for 2 critical associated complications of CKD progression, namely KRT and CVD.^41^

These costs were used to project future estimates (Supplementary Table S6), with the direct costs of CKD and KRT projected to rise by 9.3% from international $372.0 billion to $406.8 billion between 2022 and 2027 across the *Inside CKD* cohort.^38^ These rising costs correlate with an increase in the proportion of national annual health care expenditure spent on CKD and KRT (from a mean of 5.6% in 2022 to 6.4% in 2027; Supplementary Table S7). In some countries (such as India), lower costs represent scenarios in which patients paid for their treatments, rather than true decreases in public or commercial spending. These out-of-pocket costs can make care unaffordable and can hinder adherence.^54^ From 2022 to 2027, the cost of treating kidney failure increased across the *Inside CKD* cohort. Although patients undergoing KRT were projected to constitute only 5.3% of the diagnosed CKD population, these patients accounted for 45.9% of the total costs.^38^ All types of KRT were projected to increase by 2027 (by 8.5%, 12.5%, and 4.1% for hemodialysis, peritoneal dialysis, and kidney transplantation, respectively), with substantial variation between countries or regions.

Limitations of the economic burden of disease analysis include varying definitions of costs, which did not uniformly include medication and hospitalization costs in the CKD cost inputs, except for costs related to KRT.^41^ To mitigate confounding effects from comorbidities and complications, costs for CKD stages 3a–5 excluded hospitalization costs, when possible. Although partially accounted for by country-specific upper-age thresholds for KRT, conservative care was not explicitly modelled as a separate outcome by the current microsimulation and represents an important avenue for future research. *Inside CKD* did not consider indirect costs, such as lost productivity or work activity, due to insufficient data, highlighting an important evidence gap. To ensure that costs were conservative, early-stage CKD (CKD stages 1 and 2) and undiagnosed CKD were considered cost-neutral and untreated, although undiagnosed patients might be treated for complications.^41^ However, early-stage CKD, as well as advanced CKD, can be associated with excess health care spending.^55^ Therefore, *Inside CKD* likely underestimates the total economic burden of CKD.

### Which CKD Stages are Associated with the Greatest Burden of Disease?

*Inside CKD* used eGFR and albuminuria to project disease stage and progression over time, which was aligned with KDIGO recommendations to test for both parameters and to treat uACR as an independent predictor of CKD progression.^6^^,^^42^ However, evidence suggests that albuminuria testing is frequently omitted in clinical practice, possibly because of the misperception that it does not affect treatment decisions.^56^ Thus, *Inside CKD* assessed the value of uACR classification. The microsimulation projected cardiorenal complications and costs grouped by uACR in the context of decreased eGFR.^40^ Normal to mildly increased (< 30 mg/g [A1]), moderately increased (30–300 mg/g [A2]) and severely increased (> 300 mg/g [A3]) albuminuria were evaluated from 2022 to 2027, and disease progression within each uACR category was projected for each hypothetical individual.

In 2022, most of the CKD population (diagnosed and undiagnosed with any stage of CKD) was classified as uACR categories A1 or A2 (A1 = 41.3%, A2 = 49.2% and A3 = 9.6%). Normal to mildly increased albuminuria alone would not necessarily be a risk factor for poor disease outcomes. However, in the presence of declining eGFR and/or other cardiovascular risk factors, even slight increases in urinary albumin levels are concerning because albuminuria is a risk factor for CVD^57^ and/or CKD progression.^58^ Between 2022 and 2027, uACR categories A1 and A2 accounted for 96.6% of the projected cumulative incidence of CKD stage G3 to G4 transitions and for 97.5% of CKD stage G4 to G5 transitions (Supplementary Table S8). These observations align with current staging guidelines, which state that the lowest category of uACR is not associated with a lower rate of CKD progression than other uACR categories.^6^ The projected cumulative incidence of cardiorenal complications between 2022 and 2027 was also predominantly in uACR categories A1 and A2 (Supplementary Table S9) as follows: heart failure (97.6%), myocardial infarction (97.7%), stroke (96.6%), and all-cause mortality (94.9%).^40^ Notably, there were more virtual patients in the A1 and A2 groups than in the A3 group, but the risk of adverse events was higher for the A3 population. These findings support the use of recommended CKD staging that considers uACR, eGFR, and underlying risk factors.^6^

CKD trajectories are heterogeneous, so near-normal albuminuria with moderate eGFR impairment may reflect the beginning of distinct clinical journeys for different individuals. For example, an otherwise healthy 85-year-old individual with an A1 uACR category and an eGFR of 45 to 59 ml/min per 1.73 m^2^ may not progress to a more advanced disease stage over their lifetime. However, a younger patient with an A1 uACR category, CVD risk factors, and progressive eGFR decline may merit more intensive management to mitigate future adverse outcomes.^40^ In this case, there is a need for holistic CKD management tailored to the individual.

### How Would Implementing Current Recommendations for Screening Improve Diagnostic Rates?

All guidelines recommend early diagnosis and management of CKD^6^^,^^59^; however, country-specific policies to steer the identification of patients can be scarce or suboptimal.^24^^,^^60^^,^^61^ The KDIGO Controversies Conference on Early Detection and Intervention in CKD concluded that the early identification of at-risk people would be beneficial if screening programs are closely integrated with risk stratification and treatment.^62^ However, the benefit of diagnosis is only realized if health care systems fund and administer effective treatments; even lifestyle changes managed by the patient require education and support. In situations with limited resources, identifying a condition that will remain untreated offers few benefits and introduces ethical concerns for the community and care providers. In such cases, access to essential drugs and diagnostics should be secured before implementing early detection programmes.^63^ In countries where patients have access to effective treatments, screening strategies that include asymptomatic individuals with a low risk of developing CKD are not currently considered cost-effective.^62^^,^^64^ Although population-level screening for CKD remains challenging, the benefits of new treatments could make screening cost-effective in the general population.^65^^,^^66^ The US Preventive Services Task Force recently released screening recommendations that include testing at-risk populations regularly (e.g., annually for patients with diabetes or hypertension) and testing people without comorbidities who are aged ≥ 50 years (although not enough evidence was available to recommend testing frequency).^67^

*Inside CKD* modelled 2 screening strategies applied to 2 different age categories (aged ≥ 45 years and ≥ 65 years).^39^ The outputs capture the impact of screening on diagnosis rates and clinical burden, as well as the cost-effectiveness of each screening approach from 2022 to 2032. The 2 screening strategies were defined as serum creatinine tests twice a year to estimate eGFR, considered by the authors to reflect the current standard of care (strategy 1), and as an annual urinary albumin test to measure uACR, in addition to the 2 serum creatinine tests, which represents the gold-standard approach (strategy 2).

For both screening strategies, individuals were diagnosed with CKD if they received a positive test that was confirmed 3 months later. RASi treatment was assumed following diagnosis and prescribed according to national eligibility criteria; this was factored into the costs and outcomes for each virtual patient. In line with KDIGO 2012 guidelines, *Inside CKD* analyses assumed that only RASi therapy was used following diagnosis.^42^ This approach is likely to be conservative because the effects of newer, efficacious treatments, such as SGLT2is, were not included in the model. More recent guidelines, updated after the development of the *Inside CKD* microsimulation, recommend SGLT2i therapy alongside RASis for most patients with CKD.^6^ All financial evaluations were based on national cost estimates for the screening strategy and were modelled in accordance with local health care systems. Pairwise incremental cost-effectiveness ratios were derived for each strategy, which considered the overall difference between the cost of screening and treatment and the impact of any resultant diagnoses on health outcomes.

**Figure 3:**
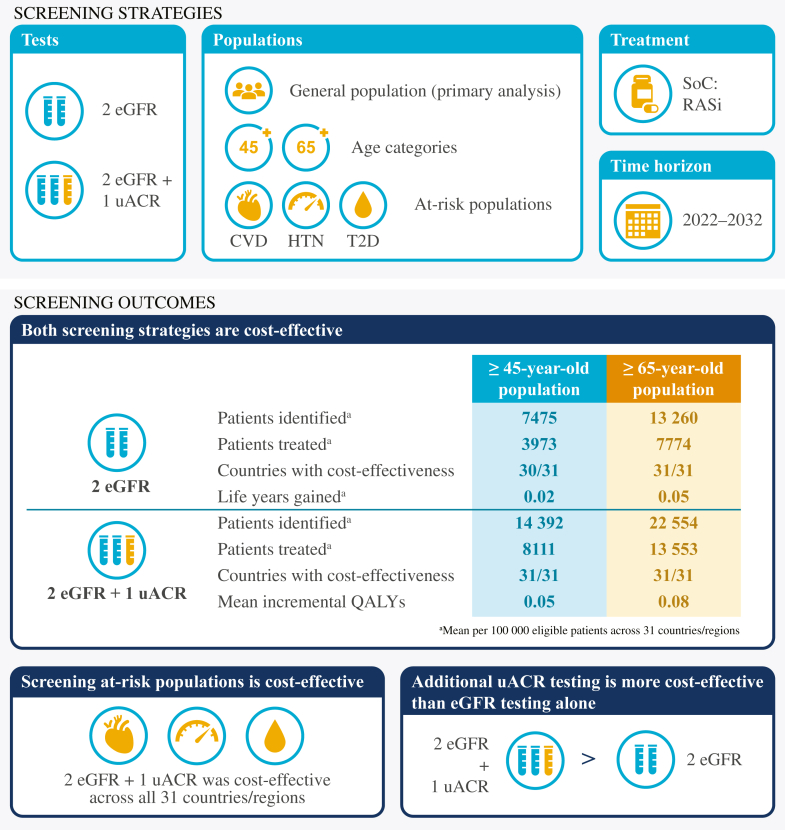
Population-level screening of CKD is predominantly cost-effective versus current practice across the *Inside CKD* cohort. The *Inside CKD* microsimulation projected CKD screening strategies in a virtual closed population across 31 countries/regions over a lifetime horizon. Populations included those aged ≥ 65 or ≥ 45 years in the general population and high-risk subgroups (patients with T2D, HTN or CVD). Modelled populations could receive two serum creatinine tests assessing eGFR (2 eGFR) or an additional uACR test (2 eGFR + 1 uACR). Eligible patients received RASis and outcomes were compared with standard of care. In line with KDIGO 2012 guidelines, *Inside CKD* assumed that only RASi therapy was used following diagnosis and that it was prescribed according to national eligibility criteria.^42^ More recent guidelines (KDIGO 2024) recommend SGLT2i therapy alongside RASis for most patients with CKD.^6^ CKD, chronic kidney disease; CVD, cardiovascular disease; eGFR, estimated glomerular filtration rate; HTN, hypertension; KDIGO, Kidney Disease: Improving Global Outcomes; QALY, quality-adjusted life year; RASi, renin-angiotensin system inhibitor; SoC, standard of care; T2D, type 2 diabetes; uACR, urine albumin–creatinine ratio. The data in this figure have been taken from Tangri *et al.*^39^ (Table 1, Figure 2, Figure 3). This work is licensed under a Creative Commons CC-BY-NC-ND license http://creativecommons.org/licenses/by/4.0/).

Both screening strategies increased the diagnosis rates for the ≥ 45 years and ≥ 65 years age populations (Figure 3 and Supplementary Table S10).^39^ Screening was associated with an increased prevalence of CKD, correlating with the high proportion of undiagnosed CKD cases in the nonscreened population. In economic terms, the early diagnosis and proactive treatment of CKD was cost-effective versus country-specific willingness-to-pay thresholds for both age groups and for both screening strategies. Increases in quality-adjusted life years were minimal. Future research could assess whether different treatment approaches (e.g., varying the proportion of patients initiating RASi and modelling the effect of SGLT2i treatment) would have yielded greater clinical and economic benefits.

A diagnosis can be valuable beyond CKD management because of the overlap in causes and risk factors with diabetes, hypertension, and CVD. Country risk profiles require careful characterization because of the variability of population factors such as obesity, smoking, and social conditions, as demonstrated by the demographic drivers for *Inside CKD.*^37^ Expanding the role of primary care in detecting CKD is a key policy discussion^68^; for instance, home-based screening has been effectively demonstrated in the Netherlands and is currently being trialed in the UK, and the targeted training of primary care teams improved early CKD detection in Italy.^69^, ^70^, ^71^ Equipping primary care teams with the means to identify and manage early-stage CKD would align with other noncommunicable disease strategies, and clinical care pathways should consider guidelines for intersecting comorbidities.^68^^,^^72^^,^^73^ Approaches linking primary and secondary health care teams have led to reduced inpatient visits, lower costs, and enriched care.^74^^,^^75^ Ideally, a referral to a nephrologist would occur during the later stages of disease or when specialist input is required. Integrated care should identify concomitant risks, especially cardiorenal risks, and increase endocrinologist and cardiologist involvement to support CKD management by nephrologists and primary care teams. Policymakers play a crucial role because health care frameworks that separate specialisms hinder the treatment of complex diseases.

## Conclusion

Current CKD management guidelines broadly align across the following 2 key areas: (i) early detection and intervention, and (ii) interdisciplinary collaboration across specialisms to manage associated comorbidities, complications, and risk factors.^59^ *Inside CKD* reinforces the importance of both areas, illustrating the high prevalence of CKD, the scale of underdiagnosis and the potential consequences. This exemplifies how current recommendations extend beyond the purview of health care professionals, requiring the support of policymakers to direct resources and to create frameworks that support integrated care.

*Inside CKD* highlights the need to raise awareness of CKD among the public, patients, health care professionals, and policymakers to increase early detection with intervention and to stimulate focused research. Like any microsimulation, *Inside CKD* relies on epidemiological data to model future outcomes, but the current data gaps are substantial. To address these gaps, nations must identify local priorities and should collaborate internationally to share knowledge.

Without health care policy changes, such as prioritizing early intervention, *Inside CKD* projects that the future global burden of CKD will be substantial. Leveraging this evidence with policymakers is an important next step in effecting change.

## Disclosure

JW reported participating in a speakers’ bureau for Akebia and Calliditas, on advisory boards for Calliditas and CSL Vifor, consulting for Akebia in the past 12 months. SMGA-G reported receiving honorarium for presentations from AstraZeneca, Bayer, and Vifor pharma; and research funding from AstraZeneca. J-MH reported fees for advisory boards or lectures from Alexion, AstraZeneca, Bayer, Boehringer Ingelheim France, Servier, and Vifor Fresenius Pharma. MJ reported the following payments to his institution: grants from AstraZeneca; consulting fees from Astellas, AstraZeneca, Bayer, Boehringer Ingelheim, CSL Vifor, GSK, and Vertex; speaker honoraria from AstraZeneca, Bayer, and Boehringer Ingelheim; and payment for expert testimony from Astellas and STADA-Eurogenerics. MJ also reports travel support from AstraZeneca and Boehringer Ingelheim and has served as volunteer cochair for KDIGO. VJ reported honoraria and consultancy fees from Visterra, Vera Therapeutics, BioCryst, Chinook, Alpine, GSK, AstraZeneca, and Boehringer Ingelheim, under the policy of all honoraria being paid to the organization. Y-SN is an employee of AstraZeneca. JFN-G reported receiving research grants from AbbVie, BioNet Medical, Boehringer Ingelheim, Sanofi, Shire, and Vifor Pharma; and honoraria for consultancies, lectures, and training activities from AbbVie, Amgen, AstraZeneca, Bayer, BioNet Medical, Boehringer Ingelheim, Eli Lilly, Esteve, Janssen, Menarini, MSD, Mundipharma, Novartis, Novo-Nordisk, Sanofi, Servier, Shire, and Vifor Pharma. JFN-G is an academic editor of the *Journal of Clinical Medicine* and associate editor of *Frontiers in Medicine*, and a member of the Scientific Advisory Board of the European Renal Association. LR is a former employee of HealthLumen and a current employee of AstraZeneca. LW is the co-founder and Chief Operating Officer of HealthLumen. CC is an employee of AstraZeneca.

## Supplementary Material

Supplementary File (PDF) **Figure S1.** Flow chart of identified, screened and included articles. **Figure S2.** Projected cumulative incidence (2022–2027) of cardiovascular complications in patients with diagnosed CKD. **Figure S3.** Projected all-cause mortality in the diagnosed and undiagnosed CKD population in 2022 per 100,000 of the CKD population. **Table S1.** Terms and limits for the search strategy. **Table S2.** Summary of key websites identified for searches. **Table S3.** PICOS criteria for article inclusion. **Table S4.** Proportion of CKD risk factors in the overall population and country/region income categories projected at baseline in 2022. **Table S5.** Prevalence of diagnosed and undiagnosed CKD cases in 2022 and 2027 per 100,000 of the national population. **Table S6.** Projected direct costs of diagnosed CKD and KRT in 2022 and 2027. **Table S7.** CKD and KRT costs as a proportion of national annual health care expenditure in 2022 and 2027. **Table S8.** Projected cumulative transitions from CKD stage G3 to G4 and G4 to G5 (kidney failure) (2022–2027) per KDIGO uACR category in the diagnosed CKD population. **Table S9.** Projected cumulative incidence of cardiorenal complications and all-cause mortality (2022–2027) per KDIGO uACR category in the diagnosed CKD population (CKD stages G3–G5) for 31 countries and regions. **Table S10.** Mean (range) diagnosis rates per 100 000 screened persons across 31 countries/region. **Useful Websites for Further Information**

• European Kidney Health Alliance: https://ekha.eu/
• Global Patient Alliance for Kidney Health – Make the change for kidney health: https://globalkidneyalliance.org/make-the-change/
• The *Inside CKD* website: https://www.insideckd.com
• International Society of Nephrology – Global Kidney Health Atlas: https://www.theisn.org/initiatives/global-kidney-health-atlas/
• Kidney Disease Improving Global Outcomes (KDIGO): https://kdigo.org/
• United States Renal Data System: https://www.niddk.nih.gov/about-niddk/strategic-plans-reports/usrds
