# Supplementary material for: “Inside CKD” Multinational-Microsimulation Modelling Insights Into the Increasing CKD Burden
Source: Kidney Int Rep. 2025 Jul 18;10(10):3356–68. doi: 10.1016/j.ekir.2025.07.014 (PMC12546715; doi:10.1016/j.ekir.2025.07.014)
Supplement: Supplementary File (PDF) — Figure S1. Flow chart of identified, screened and included articles. Figure S2. Projected cumulative incidence (2022–2027) of cardiovascular complications in patients with diagnosed CKD. Figure S3. Projected all-cause mortality in the diagnosed and undiagnosed CKD population in 2022 per 100,000 of the CKD population. Table S1. Terms and limits for the search strategy. Table S2. Summary of key websites identified for searches. Table S3. PICOS criteria for article inclusion. Table S4. Proportion of CKD risk factors in the overall population and country/region income categories projected at baseline in 2022. Table S5. Prevalence of diagnosed and undiagnosed CKD cases in 2022 and 2027 per 100,000 of the national population. Table S6. Projected direct costs of diagnosed CKD and KRT in 2022 and 2027. Table S7. CKD and KRT costs as a proportion of national annual health care expenditure in 2022 and 2027. Table S8. Projected cumulative transitions from CKD stage G3 to G4 and G4 to G5 (kidney failure) (2022–2027) per KDIGO uACR category in the diagnosed CKD population. Table S9. Projected cumulative incidence of cardiorenal complications and all-cause mortality (2022–2027) per KDIGO uACR category in the diagnosed CKD population (CKD stages G3–G5) for 31 countries and regions. Table S10. Mean (range) diagnosis rates per 100 000 screened persons across 31 countries/region. Useful Websites for Further Information•European Kidney Health Alliance: https://ekha.eu/•Global Patient Alliance for Kidney Health – Make the change for kidney health: https://globalkidneyalliance.org/make-the-change/•The Inside CKD website: https://www.insideckd.com•International Society of Nephrology – Global Kidney Health Atlas: https://www.theisn.org/initiatives/global-kidney-health-atlas/•Kidney Disease Improving Global Outcomes (KDIGO): https://kdigo.org/•United States Renal Data System: https://www.niddk.nih.gov/about-niddk/strategic-plans-reports/usrds [file mmc1.pdf]

'Inside CKD' Multinational-microsimulation Modelling Insights into the Increasing CKD Burden

Supplementary Material

Contents

Figure S1. Flow chart of identified, screened and included articles 1
Figure S2. Projected cumulative incidence (2022–2027) of cardiovascular complications in patients with diagnosed CKD 2
Figure S3. Projected all-cause mortality in the diagnosed and undiagnosed CKD population in 2022 per 100 000 of the CKD population 4
Table S1. Terms and limits for the search strategy 6
Table S2. Summary of key websites identified for searches 7
Table S3. PICOS criteria for article inclusion 8
Table S4. Proportion of CKD risk factors in the overall population and country/region income categories projected at baseline in 2022 9
Table S5. Prevalence of diagnosed and undiagnosed CKD cases in 2022 and 2027 per 100 000 of the national population 11
Table S6. Projected direct costs of diagnosed CKD and KRT in 2022 and 2027 14
Table S7. CKD and KRT costs as a proportion of national annual healthcare expenditure in 2022 and 2027 16
Table S8. Projected cumulative transitions from CKD stage G3 to G4 and G4 to G5 (kidney failure) (2022–2027) per KDIGO uACR category in the diagnosed CKD population 19
Table S9. Projected cumulative incidence of cardiorenal complications and all-cause mortality (2022–2027) per KDIGO uACR category in the diagnosed CKD population (CKD stages G3–G5) for 31 countries and regions 21
Table S10. Mean (range) diagnosis rates per 100 000 screened persons across 31 countries/regions 23

(PDF) Supplementary information is available at KI Report's website.

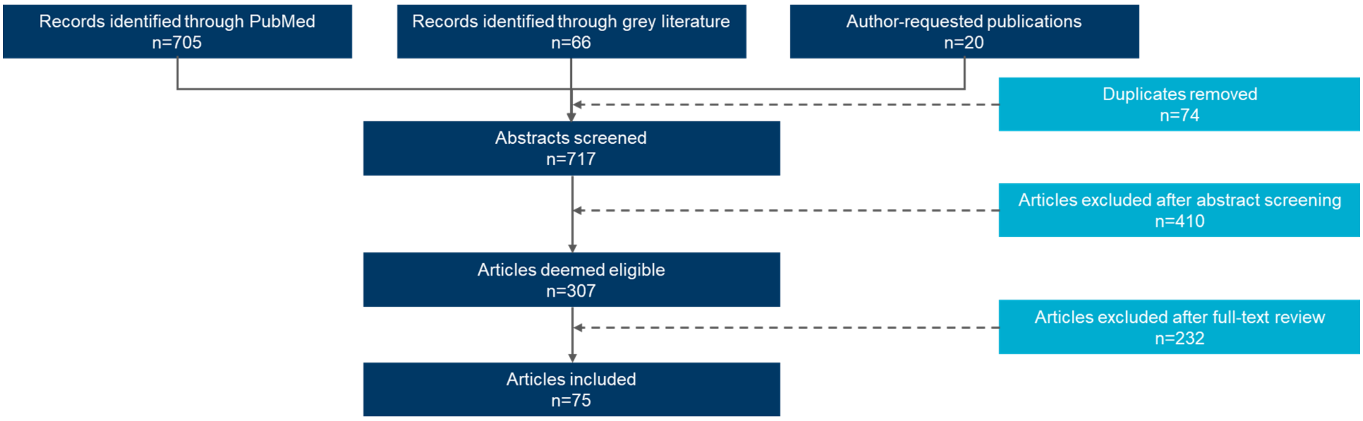

Figure S1. Flow chart of identified, screened and included articles

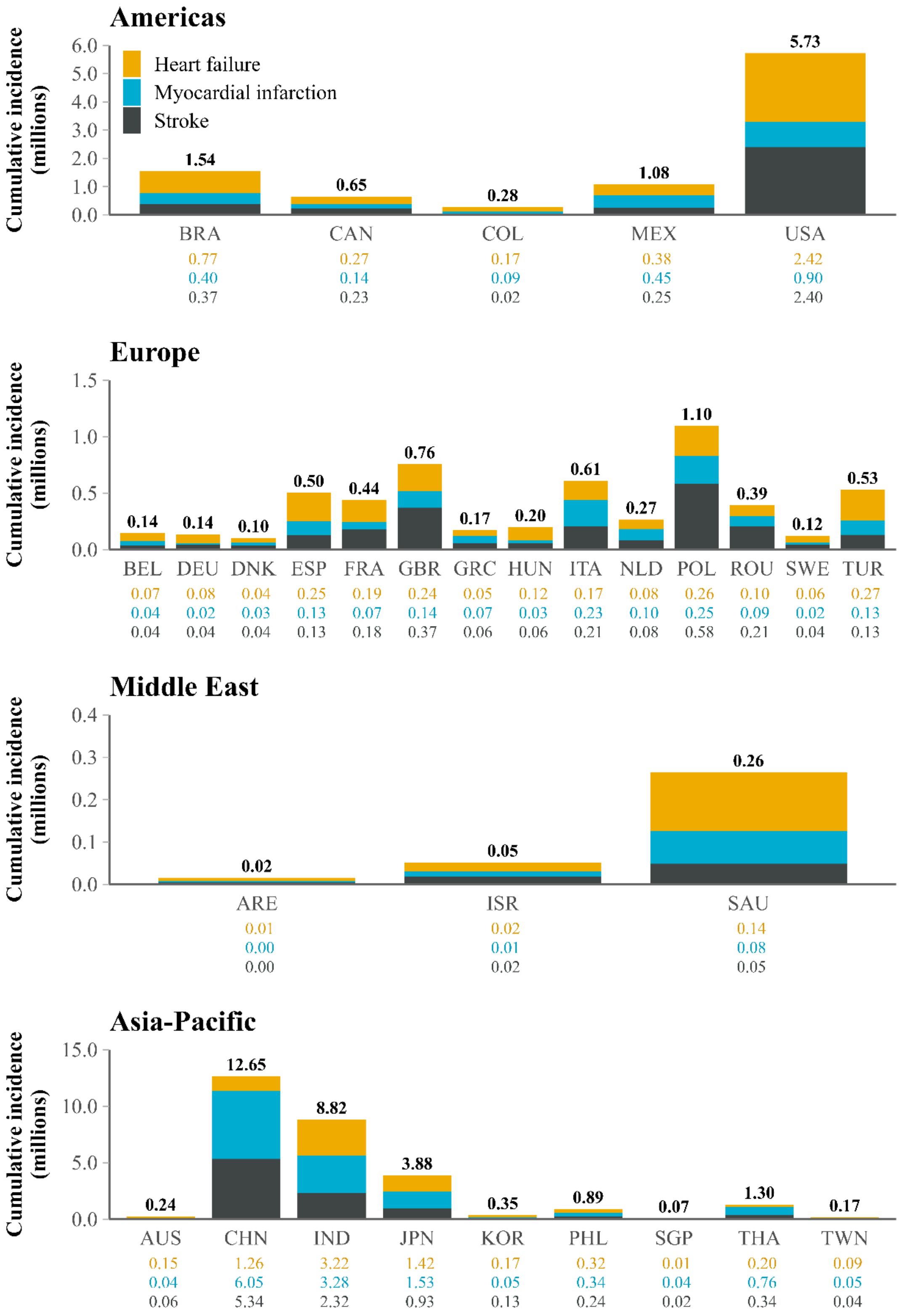

Figure S2. Projected cumulative incidence (2022–2027) of cardiovascular complications in patients with diagnosed CKD

ARE, United Arab Emirates; AUS, Australia; BEL, Belgium; BRA, Brazil; CAN, Canada; CHN, China; CKD, chronic kidney disease; COL, Colombia; DEU, Germany; DNK, Denmark; ESP, Spain; FRA, France; GBR, United Kingdom; GRC, Greece; HUN, Hungary; IND, India; ISR, Israel; ITA, Italy; JPN, Japan; KOR, South

Korea; MEX, Mexico; NLD, Netherlands; PHL, Philippines; POL, Poland; ROU, Romania; SAU, Saudi Arabia; SGP, Singapore; SWE, Sweden; THA, Thailand; TUR, Türkiye; TWN, Taiwan.

Note: The United Arab Emirates has a large and diverse Expatriate population with a different CKD profile; only the Emirati population has been presented here.

CC-BY 2024. Projecting the clinical burden of chronic kidney disease at the patient level (Inside CKD): a microsimulation modelling study. G. Chertow *et al.* (Figure 3) https://doi.org/10.1016/j.eclinm.2024.102614.[37] This work is licensed under a CC-BY licence http://creativecommons.org/licenses/by/4.0/.

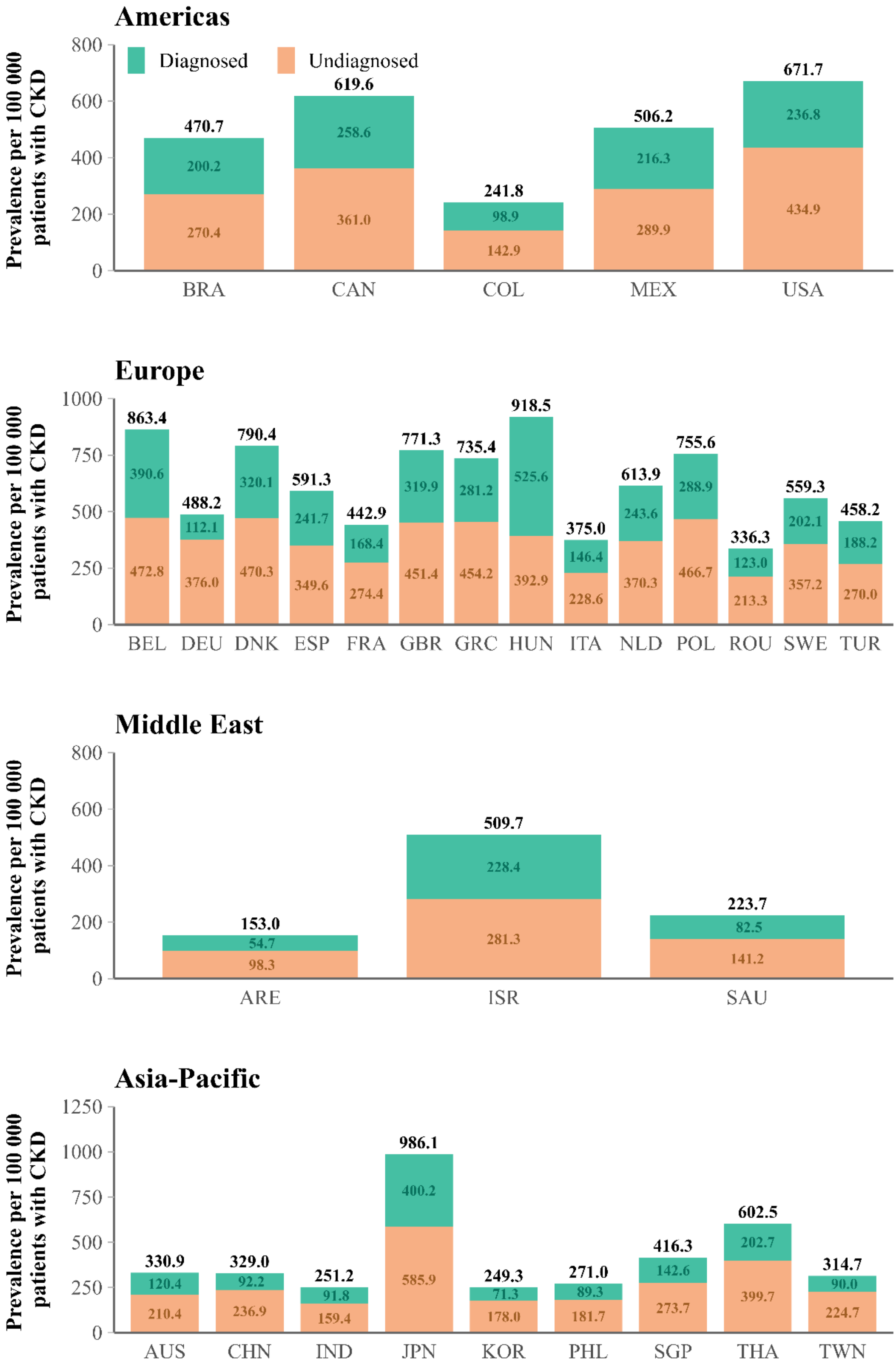

Figure S3. Projected all-cause mortality in the diagnosed and undiagnosed CKD population in 2022 per 100 000 of the CKD population

ARE, United Arab Emirates; AUS, Australia; BEL, Belgium; BRA, Brazil; CAN, Canada; CHN, China; CKD, chronic kidney disease; COL, Colombia; DEU, Germany; DNK, Denmark; ESP, Spain; FRA, France; GBR,

United Kingdom; GRC, Greece; HUN, Hungary; IND, India; ISR, Israel; ITA, Italy; JPN, Japan; KOR, South Korea; MEX, Mexico; NLD, Netherlands; PHL, Philippines; POL, Poland; ROU, Romania; SAU, Saudi Arabia; SGP, Singapore; SWE, Sweden; THA, Thailand; TUR, Türkiye; TWN, Taiwan.

Note: The United Arab Emirates has a large and diverse Expatriate population with a different CKD profile; only the Emirati population has been presented here.

CC-BY 2024. Projecting the clinical burden of chronic kidney disease at the patient level (Inside CKD): a microsimulation modelling study. G. Chertow *et al.* (Figure 5a) https://doi.org/10.1016/j.eclinm.2024.102614.[37] This work is licensed under a CC-BY licence http://creativecommons.org/licenses/by/4.0/.

Table S1. Terms and limits for the search strategy

Primary search string	Secondary search string(s)	Number of results
(“chronic kidney disease" OR "ckd" OR “chronic renal” OR "renal replacement")	AND ("global burden")	105
	AND ("management guidelines")	20
	AND ("call to action")	23
	AND (“registry data” OR “claims data”)	66
	AND ("policy")	491
**Limitations**		
Article language	English	
Species	Human	
Article types	Books and documents; Clinical conference; Comment; Congress; Editorial; Observational study; Practice guideline; Review; Systematic review; Guideline; Government publication; Meta-analysis	

CKD, chronic kidney disease.

Table S2. Summary of key websites identified for searches

**Websites retrieved and manually searched**		
American Society of Nephrology (https://www.asn-online.org/)	Canadian national guidelines	Centers for Disease Control and Prevention (https://www.cdc.gov/index.htm)
China Kidney Network (https://www.chinakidney.net/index.php/welcome.html)	CKD-DOPPS (https://www.dopps.org/OurStudies/ChronicKidneyDiseaseCKDopps.aspx)	European Renal Association (https://www.era-online.org/)
Institute for Health Metrics and Evaluation: GBD 2019 (https://www.healthdata.org/research-analysis/gbd)	International Society of Nephrology (https://www.theisn.org/): ISN Atlas reports and other key documents	Kidney Disease Improving Global Outcomes (https://kdigo.org/)
National Institute for Health and Care Excellence (https://www.nice.org.uk/)	National Institute of Health (https://www.nih.gov/)	National Institute of Diabetes and Digestive and Kidney Diseases (https://www.niddk.nih.gov/)
National Kidney Foundation (https://www.kidney.org/)	The Asian Pacific Society of Nephrology (https://apsneph.org/web/index.php)	The Renal Physicians Association (https://www.renalmd.org/)
UK Kidney Association (https://ukkidney.org/)	United Nations (https://www.un.org/en/)	United States Renal Data System (https://usrds-adr.niddk.nih.gov/2022)
World Health Organization (https://www.who.int/)<br>Including all regional offices: https://www.afro.who.int/; https://www.paho.org/en; https://www.who.int/southeastasia; https://www.who.int/westernpacific; https://www.emro.who.int/index.html; https://www.who.int/europe/home?v=welcomev		

Table S3. PICOS criteria for article inclusion

Criteria	Full inclusion criteria
**Population**	People with CKD
**Interventions/comparators**	CKD healthcare policies; CKD treatment/management guidelines; CKD standard-of-care/screening programmes
**Outcomes**	Global disease epidemiology; international guidelines, policy, or management recommendations; international or national (for one or more of the 31 *Inside CKD* countries) registry recommendations
**Study design/type**	Epidemiological multinational/global studies, clinical guidelines, qualitative articles of international influence (e.g. commentaries/calls to action), government publications, international governing body publications, systematic/targeted literature reviews

CKD, chronic kidney disease; PICOS, Population, Intervention, Comparison, Outcome and Study design.

Table S4. Proportion of CKD risk factors in the overall population and country/region income categories projected at baseline in 2022

Country/region	Population aged ≥ 65 years (%)	Hypertension (%)	Coronary heart disease (%)	Stroke (%)	Diabetes (%)	Smoking (%)	Obesity (%)	Income category
**Americas**								
**Brazil**	9.6	23.3	1.9	1.4	10.1	16.5	22.1	Middle
**Canada**	18.1	13.2	3.3	1.8	8.4	17.5	29.4	High
**Colombia**	9.1	19.2	2.1	1.0	9.8	7.9	22.3	Middle
**Mexico**	7.6	19.7	2.0	1.1	15.6	13.9	28.9	Middle
**USA**	16.6	12.9	2.9	2.3	9.4	25.1	36.2	High
**Europe**								
**Belgium**	19.3	17.5	3.8	1.3	4.8	25.0	22.1	High
**Denmark**	20.2	20.6	3.6	1.3	5.6	18.6	19.7	High
**France**	20.8	22.0	3.4	1.2	5.4	34.6	21.6	High
**Germany**	21.7	19.9	4.5	1.6	5.3	28.0	22.3	High
**Greece**	22.3	19.1	3.7	1.6	5.1	39.1	24.9	High
**Hungary**	20.2	30.0	6.9	2.3	6.0	30.6	26.4	High
**Italy**	23.3	21.2	4.7	1.3	5.1	23.4	19.9	High
**Netherlands**	20.0	18.7	4.6	1.3	5.3	23.4	20.4	High
**Poland**	18.7	28.7	4.1	1.7	9.0	26.0	23.1	High
**Romania**	19.2	30.6	5.8	2.4	7.7	25.5	22.5	Middle
**Spain**	20.0	19.2	3.5	1.3	6.3	27.9	23.8	High
**Sweden**	20.3	19.3	3.8	1.8	4.2	28.8	20.6	High
**Türkiye**	9.0	20.3	3.6	1.4	7.9	29.3	32.1	Middle
**UK**	18.7	15.2	3.1	1.1	5.2	19.2	27.8	High
**Middle East**								
**Israel**	12.4	16.6	2.2	0.8	7.4	25.5	26.1	High
**Saudi Arabia**	3.5	23.3	2.4	1.4	19.6	16.6	35.4	High
**UAE**	1.3	21.1	2.2	1.5	18.8	18.2	31.7	High
**Asia-Pacific**								
**Australia**	16.2	15.2	5.3	1.1	6.6	16.2	29.0	High
**China**	12.0	19.2	3.3	2.1	8.8	24.7	6.2	Middle
**India**	6.6	25.8	2.8	0.7	9.0	27.0	3.9	Middle
**Japan**	28.4	17.6	3.0	3.3	7.7	21.9	4.3	High
**Philippines**	5.5	22.6	1.7	1.2	9.7	24.3	6.4	Middle
**Singapore**	13.4	14.6	2.0	1.5	9.5	16.5	6.1	High
**South Korea**	15.8	11.0	1.6	2.0	7.5	22.0	4.7	High
**Taiwan**	16.1	25.0	3.7	1.8	8.2	19.4	22.8	High
**Thailand**	13.0	22.3	2.3	1.7	7.5	22.8	10.0	High
**Mean**								
**High income**	17.4	19.3	3.5	1.6	7.7	23.8	22.1	N/A
**Middle income**	9.8	22.6	2.9	1.4	9.8	21.1	18.1	N/A

CKD, chronic kidney disease.

CC-BY 2024. Projecting the clinical burden of chronic kidney disease at the patient level (Inside CKD): a microsimulation modelling study. G. Chertow *et al.* (Table S4) https://doi.org/10.1016/j.eclinm.2024.102614.[37] This work is licensed under a CC-BY licence http://creativecommons.org/licenses/by/4.0/

Table S5. Prevalence of diagnosed and undiagnosed CKD cases in 2022 and 2027 per 100 000 of the national population

Diagnosed CKD prevalence per 100 000 people (including KRT)									
Country/region	Year	CKD stage						TOTAL	% change* 2022–2027
		1	2	3a	3b	4	5		
**Americas**									
**Brazil**	**2022**	67	469	1379	888	139	20	**2963**	7.8
	**2027**	74	430	1677	783	176	55	**3193**	
**Canada**	**2022**	133	652	2057	741	256	46	**3885**	1.1
	**2027**	144	598	2246	646	203	88	**3926**	
**Colombia**	**2022**	76	322	915	304	85	11	**1713**	22.3
	**2027**	88	295	1365	249	68	31	**2095**	
**Mexico**	**2022**	190	870	658	418	158	131	**2425**	0.8
	**2027**	215	745	924	364	136	63	**2446**	
**USA**	**2022**	229	1036	1268	756	98	17	**3404**	−0.3
	**2027**	252	896	1373	663	162	48	**3394**	
**Europe**									
**Belgium**	**2022**	128	657	2081	871	595	131	**4463**	−5.3
	**2027**	141	592	2280	711	340	164	**4228**	
**Denmark**	**2022**	134	673	2233	766	243	37	**4087**	−2.6
	**2027**	151	601	2494	529	137	69	**3981**	
**France**	**2022**	155	635	1244	431	241	111	**2817**	7.3
	**2027**	173	528	1647	356	217	102	**3023**	
**Germany**	**2022**	359	445	739	243	83	14	**1884**	11.1
	**2027**	349	448	991	209	73	24	**2093**	
**Greece**	**2022**	92	163	2328	544	186	32	**3345**	11.3
	**2027**	106	148	2865	431	114	60	**3723**	
**Hungary**	**2022**	136	683	1749	1021	242	28	**3858**	−7.1
	**2027**	153	599	2021	640	119	54	**3585**	
**Italy**	**2022**	88	349	724	362	166	89	**1778**	6.3
	**2027**	94	288	976	352	118	62	**1890**	
**Netherlands**	**2022**	134	754	1720	551	181	28	**3369**	7.7
	**2027**	151	676	2242	392	116	50	**3628**	
**Poland**	**2022**	89	688	1682	523	159	24	**3165**	7.5
	**2027**	107	641	2177	345	89	42	**3401**	
**Romania**	**2022**	61	332	1384	231	49	6	**2063**	33.2
	**2027**	69	262	2160	170	48	39	**2748**	
**Spain**	**2022**	71	493	2084	522	180	37	**3387**	9.5
	**2027**	79	439	2456	525	141	69	**3709**	
**Sweden**	**2022**	458	385	702	554	222	165	**2486**	−3.6
	**2027**	517	321	911	381	141	126	**2398**	
**Türkiye**	**2022**	230	740	729	555	173	75	**2502**	0.4
	**2027**	253	667	1034	348	161	49	**2512**	
**UK**	**2022**	128	634	2086	776	267	48	**3940**	−3.2
	**2027**	140	567	2202	641	188	75	**3814**	
**Middle East**									
**Israel**	**2022**	401	202	792	527	329	67	**2319**	−9.4
	**2027**	420	185	902	375	160	60	**2101**	
**Saudi Arabia**	**2022**	179	720	1131	593	302	284	**3210**	15.0
	**2027**	202	673	1441	687	333	355	**3692**	
**UAE (Emirati)**	**2022**	173	678	1051	471	279	235	**2888**	20.1
	**2027**	205	724	1563	457	303	217	**3469**	
**UAE (Expatriate)** †	**2022**	192	100	91	24	5	7	**419**	28.8
	**2027**	195	102	209	22	9	3	**540**	
**Asia-Pacific**									
**Australia**	**2022**	152	604	896	216	103	58	**2030**	7.0
	**2027**	168	516	1141	219	82	46	**2171**	
**China**	**2022**	267	263	432	84	37	16	**1098**	−1.6
	**2027**	270	248	455	80	19	8	**1081**	
**India**	**2022**	151	190	310	111	35	48	**846**	−2.5
	**2027**	157	172	401	67	19	8	**825**	
**Japan**	**2022**	94	1717	1999	1710	370	36	**5927**	9.9
	**2027**	111	1518	3179	1122	487	97	**6514**	
**Philippines**	**2022**	276	137	713	126	48	30	**1331**	6.4
	**2027**	295	117	820	134	28	22	**1417**	

**Singapore**	**2022**	859	564	1617	312	141	89	**3583**	10.6
	**2027**	920	546	1888	401	113	95	**3964**	
**South Korea**	**2022**	197	771	951	246	119	22	**2305**	32.3
	**2027**	212	699	1628	286	162	64	**3051**	
**Taiwan**	**2022**	196	749	935	242	114	21	**2258**	28.8
	**2027**	208	658	1574	266	146	56	**2908**	
**Thailand**	**2022**	356	523	2864	743	205	206	**4897**	11.2
	**2027**	407	472	3142	895	263	269	**5448**	

Undiagnosed CKD prevalence per 100 000 people (including KRT)									
**Country/region**	**Year**	**CKD stage**						**TOTAL**	**% change[*] 2022–2027**
		**1**	**2**	**3a**	**3b**	**4**	**5**		
**Americas**									
**Brazil**	**2022**	1503	1263	3318	661	17	1	**6763**	10.3
	**2027**	1664	1149	4042	581	21	2	**7459**	
**Canada**	**2022**	2963	1743	3535	1125	94	0	**9460**	2.8
	**2027**	3216	1598	3852	983	74	0	**9723**	
**Colombia**	**2022**	1668	867	2194	227	10	0	**4966**	12.0
	**2027**	1859	793	2714	184	8	1	**5560**	
**Mexico**	**2022**	4265	2322	1581	309	19	6	**8502**	7.9
	**2027**	4725	1947	2212	268	16	3	**9171**	
**USA**	**2022**	5098	2774	3043	561	12	1	**11 488**	1.9
	**2027**	5526	2373	3294	493	19	2	**11 708**	
**Europe**									
**Belgium**	**2022**	2847	1758	3571	1332	215	0	**9723**	1.1
	**2027**	3128	1577	3917	1082	123	0	**9827**	
**Denmark**	**2022**	2913	1803	3824	1172	89	0	**9801**	−2.0
	**2027**	3228	1605	3910	812	49	0	**9605**	
**France**	**2022**	3429	1692	2134	657	85	2	**7999**	4.9
	**2027**	3795	1407	2578	535	72	2	**8389**	
**Germany**	**2022**	7967	1193	1269	366	30	0	**10 825**	1.4
	**2027**	7719	1206	1707	315	26	0	**10 972**	
**Greece**	**2022**	1535	1820	3989	825	67	0	**8236**	3.7
	**2027**	1766	1642	4443	652	41	0	**8544**	
**Hungary**	**2022**	2971	1819	4198	753	29	1	**9771**	−1.0
	**2027**	3252	1590	4341	472	14	2	**9671**	
**Italy**	**2022**	1974	933	1740	267	20	4	**4938**	11.6
	**2027**	2118	771	2345	259	14	3	**5509**	
**Netherlands**	**2022**	2952	2021	2947	840	65	0	**8825**	4.2
	**2027**	3263	1812	3476	599	42	0	**9192**	
**Poland**	**2022**	1961	1856	2889	802	57	0	**7565**	4.6
	**2027**	2348	1724	3285	526	32	0	**7916**	
**Romania**	**2022**	1326	889	2361	358	19	0	**4952**	14.6
	**2027**	1468	703	3225	262	18	0	**5676**	
**Spain**	**2022**	1582	1329	3569	797	65	0	**7342**	9.0
	**2027**	1764	1177	4214	794	52	0	**8001**	
**Sweden**	**2022**	1830	1537	2803	548	54	0	**6772**	3.4
	**2027**	2060	1277	3256	377	32	0	**7002**	
**Türkiye**	**2022**	5094	1982	1254	847	62	0	**9239**	5.7
	**2027**	5604	1789	1778	534	56	0	**9761**	
**UK**	**2022**	2872	1693	3589	1184	98	0	**9437**	0.3
	**2027**	3125	1513	3788	974	69	0	**9468**	
**Middle East**									
**Israel**	**2022**	6792	2263	1450	275	31	0	**10 811**	1.5
	**2027**	7047	2074	1644	197	16	0	**10 978**	
**Saudi Arabia**	**2022**	3955	1907	2725	437	37	12	**9074**	12.2
	**2027**	4404	1754	3455	510	40	16	**10 179**	
**UAE (Emirati)**	**2022**	3764	1827	2482	343	32	10	**8457**	11.5
	**2027**	4169	1890	3012	318	31	9	**9429**	
**UAE (Expatriate)[†]**	**2022**	4199	271	222	18	1	0	**4711**	4.8
	**2027**	4272	268	377	16	1	0	**4934**	
**Asia-Pacific**									
**Australia**	**2022**	3374	1614	1535	327	37	1	**6888**	6.5
	**2027**	3659	1364	1953	331	29	1	**7338**	
	**2022**	5862	701	741	128	13	0	**7446**	

**China**	**2027**	5950	661	775	123	7	0	**7516**	0.9
**India**	**2022**	3389	509	531	168	12	0	**4610**	3.4
	**2027**	3518	455	684	102	7	0	**4766**	
**Japan**	**2022**	2086	4609	3434	2599	133	0	**12 860**	7.9
	**2027**	2452	4071	5464	1714	174	0	**13 875**	
**Philippines**	**2022**	1608	792	1440	254	44	0	**4137**	5.3
	**2027**	1721	681	1660	270	25	0	**4356**	
**Singapore**	**2022**	5005	3279	3271	628	126	0	**12 309**	7.7
	**2027**	5358	3172	3812	811	102	0	**13 255**	
**South Korea**	**2022**	4394	2056	1633	376	44	0	**8503**	16.2
	**2027**	4726	1867	2796	431	59	0	**9879**	
**Taiwan**	**2022**	4345	2003	1604	370	41	0	**8362**	14.0
	**2027**	4606	1762	2703	408	54	0	**9532**	
**Thailand**	**2022**	2071	3039	5777	1497	183	0	**12 568**	7.3
	**2027**	2367	2745	6337	1802	235	0	**13 486**	

CKD, chronic kidney disease; KRT, kidney replacement therapy.

*Note: individual values have been rounded to whole numbers. Slight discrepancies may occur between the total sum reported as a result.

†UAE expatriate population was modelled separately from the Emirati population owing to distinct population demographics requiring separate input data.

CC-BY 2024. Projecting the clinical burden of chronic kidney disease at the patient level (Inside CKD): a microsimulation modelling study. G. Chertow *et al.* (Table 3) https://doi.org/10.1016/j.eclinm.2024.102614.[37] This work is licensed under a CC-BY licence http://creativecommons.org/licenses/by/4.0/.

Table S6. Projected direct costs of diagnosed CKD and KRT in 2022 and 2027

Country/region		Total direct costs of diagnosed CKD and KRT				
		2022		2027		Change in total cost 2022–2027, %
		Total cost US$, billion	Cost per capita, US$ per person*	Total cost US$, billion	Cost per capita, US$ per person*	
Americas	Brazil	9.77	45.38	11.59	52.42	18.61
	Canada	7.38	192.20	8.09	202.53	9.63
	Colombia	1.76	34.23	2.19	41.63	24.00
	Mexico	5.97	45.35	6.38	46.33	6.88
	USA†	161.95	483.70	173.41	503.96	7.08
Europe	Belgium	2.84	243.50	2.86	242.12	0.70
	Denmark	0.48	81.48	0.53	88.75	10.94
	France	14.52	221.39	17.93	270.29	23.47
	Germany	12.34	147.16	13.81	165.67	11.85
	Greece	1.29	124.77	1.46	145.42	13.59
	Hungary	0.85	88.46	0.87	92.16	2.47
	Italy	5.94	98.50	6.08	102.04	2.38
	Netherlands	1.80	104.59	2.02	115.98	11.97
	Poland	1.41	37.34	1.73	46.48	23.10
	Romania	1.35	70.73	1.84	99.01	36.75
	Spain	7.15	149.46	8.14	167.90	13.81
	Sweden	1.25	122.79	1.26	120.36	0.61
	Türkiye	4.58	53.51	5.04	57.55	10.12
	UK	4.48	65.43	4.71	67.48	5.04
Middle East	Israel	1.55	173.22	1.53	159.41	-1.16
	Saudi Arabia	6.50	181.37	8.71	228.56	34.00
	UAE (Emirati)	0.31	190.19	0.38	216.87	24.33
	UAE (Expatriate)‡	0.03	3.68	0.06	7.18	100.00
Asia-Pacific	Australia	2.35	90.18	2.66	96.94	13.02
	China	45.07	31.11	45.21	30.93	0.33
	India†	15.87	11.28	16.17	11.01	1.90
	Japan	33.76	268.83	38.31	312.05	13.46
	Philippines	1.93	17.15	2.17	18.16	12.58
	Singapore	1.07	179.60	1.29	209.89	21.09
	South Korea	6.90	134.49	8.45	164.67	22.35
	Taiwan	3.47	145.38	4.16	173.20	19.68
	Thailand	6.12	87.26	7.75	110.05	26.68
	**Sum**	**372.03**		**406.78**		

CKD, chronic kidney disease; KRT, kidney replacement therapy.
*Cost per capita represents the total cost divided by the entire general population.
†In settings without a single public healthcare system, the model was adapted to use a commercial or equivalent framework, or a mixed funding model. For example, in the case of the USA, costs were split into Medicare and commercial categories; for India, costs were split into four components: charitable, private, employment insurance and public.

‡UAE expatriate population was modelled separately from the Emirati population owing to distinct population demographics requiring separate input data.

CC-BY 2024. Projecting the economic burden of chronic kidney disease at the patient level (Inside CKD): a microsimulation modelling study. S. Chadban *et al*. (Table S3). https://doi.org/10.1016/j.eclinm.2024.102615.[38] This work is licensed under a CC-BY licence http://creativecommons.org/licenses/by/4.0/).

Table S7. CKD and KRT costs as a proportion of national annual healthcare expenditure in 2022 and 2027

Country/region		Year	Proportion of national annual healthcare expenditure* (%)							
			CKD stage				KRT			Sum
			3a	3b	4	5	Haemo-dialysis	Peritoneal dialysis	Transplant	
Americas	**Brazil**	**2022**	8.59	6.23	1.28	0.17	5.00	0.42	0.77	**22.44**
		**2027**	10.72	5.64	1.65	0.45	6.49	0.68	0.99	**26.62**
	**Canada**	**2022**	1.86	0.94	0.52	0.19	0.80	0.12	0.27	**4.71**
		**2027**	2.12	0.86	0.43	0.38	0.95	0.15	0.27	**5.16**
	**Colombia**	**2022**	1.16	0.39	0.15	0.01	1.37	0.50	0.14	**3.72**
		**2027**	1.76	0.32	0.12	0.04	1.55	0.61	0.21	**4.61**
	**Mexico**	**2022**	1.87	1.19	0.49	1.09	2.00	1.12	1.05	**8.81**
		**2027**	2.75	1.08	0.44	0.48	2.46	1.19	1.01	**9.42**
	**USA**[†]	**2022**	0.97	0.78	0.12	0.01	1.38	0.14	0.33	**3.72**
		**2027**	0.98	0.68	0.18	0.04	1.44	0.15	0.34	**3.82**
Europe	**Belgium**	**2022**	1.56	0.67	0.81	0.28	1.45	0.08	0.16	**5.03**
		**2027**	1.73	0.56	0.47	0.38	1.63	0.09	0.20	**5.06**
	**Denmark**	**2022**	0.55	0.21	0.09	0.01	0.49	0.08	0.16	**1.60**
		**2027**	0.63	0.15	0.05	0.03	0.61	0.11	0.19	**1.77**
	**France**	**2022**	1.10	0.39	0.38	0.00	2.67	0.12	0.62	**5.29**
		**2027**	1.48	0.33	0.33	0.00	3.45	0.17	0.78	**6.54**
	**Germany**	**2022**	0.84	0.28	0.11	0.03	1.39	0.07	0.10	**2.81**
		**2027**	1.11	0.23	0.10	0.06	1.46	0.07	0.11	**3.15**
	**Greece**	**2022**	0.54	0.13	0.14	0.02	2.51	0.19	0.15	**3.67**
		**2027**	0.65	0.10	0.08	0.04	2.91	0.22	0.17	**4.17**
	**Hungary**	**2022**	1.28	0.86	0.23	0.02	1.41	0.15	1.36	**5.30**
		**2027**	1.45	0.53	0.11	0.04	1.48	0.16	1.67	**5.43**
	**Italy**	**2022**	0.54	0.39	0.30	0.08	1.22	0.16	0.39	**3.09**
		**2027**	0.72	0.38	0.21	0.06	1.25	0.17	0.37	**3.16**
	**Netherlands**	**2022**	0.36	0.13	0.06	0.01	0.68	0.13	0.36	**1.72**
		**2027**	0.47	0.09	0.04	0.02	0.78	0.16	0.37	**1.93**
	**Poland**	**2022**	0.04	0.04	0.03	0.00	1.70	0.10	0.19	**2.09**
		**2027**	0.05	0.03	0.02	0.00	2.12	0.13	0.23	**2.58**
	**Romania**	**2022**	2.13	0.41	0.10	0.01	3.44	0.05	0.10	**6.24**
		**2027**	3.24	0.29	0.09	0.05	4.09	0.06	0.71	**8.53**
	**Spain**	**2022**	1.57	1.23	0.43	0.08	1.69	0.22	0.30	**5.52**
		**2027**	1.88	1.26	0.34	0.14	2.02	0.29	0.36	**6.29**

	**Sweden**	**2022**	0.38	0.49	0.29	0.01	0.55	0.10	0.20	**2.02**
		**2027**	0.51	0.34	0.17	0.04	0.61	0.12	0.24	**2.03**
	**Türkiye**	**2022**	1.33	1.02	0.86	0.17	1.79	0.22	0.37	**5.76**
		**2027**	1.94	0.65	0.82	0.13	2.16	0.31	0.33	**6.34**
	**UK**	**2022**	0.81	0.34	0.15	0.04	0.43	0.06	0.20	**2.02**
		**2027**	0.87	0.29	0.11	0.06	0.50	0.09	0.20	**2.12**
**Middle East**	**Israel**	**2022**	1.24	0.82	0.60	0.19	1.54	0.11	0.08	**4.57**
		**2027**	1.51	0.63	0.31	0.22	1.65	0.12	0.08	**4.52**
	**Saudi Arabia**	**2022**	2.41	1.26	0.75	0.81	0.96	0.11	0.24	**6.53**
		**2027**	3.26	1.56	0.88	0.84	1.55	0.21	0.45	**8.76**
	**UAE (Emirati)**	**2022**	0.17	0.11	0.10	0.02	0.74	0.05	0.11	**1.29**
		**2027**	0.28	0.12	0.11	0.01	0.89	0.05	0.15	**1.60**
	UAE (Expatriate)[‡]	***2022***	0.07	0.03	0.01	0.01	0.02	0.001	0.01	**0.15**
		2027	0.18	0.03	0.02	0.003	0.04	0.003	0.004	**0.27**
**Asia-Pacific**	**Australia**	**2022**	0.70	0.17	0.33	0.10	0.87	0.13	0.19	**2.49**
		**2027**	0.93	0.18	0.28	0.09	0.98	0.15	0.19	**2.81**
	**China**	**2022**	1.21	0.23	0.29	0.04	1.25	0.11	0.14	**3.27**
		**2027**	1.29	0.23	0.15	0.02	1.34	0.12	0.14	**3.28**
	**India**[†]	**2022**	8.44	3.04	0.88	1.44	1.01	0.07	1.13	**16.00**
		**2027**	11.40	1.92	0.50	0.25	1.00	0.08	1.16	**16.31**
	**Japan**	**2022**	1.46	1.25	0.76	0.05	3.72	0.08	0.03	**7.36**
		**2027**	2.28	0.80	0.98	0.15	4.02	0.09	0.04	**8.35**
	**Philippines**	**2022**	0.81	0.18	0.11	0.20	3.20	0.07	0.10	**4.67**
		**2027**	0.99	0.21	0.07	0.14	3.67	0.08	0.09	**5.25**
	**Singapore**	**2022**	1.87	0.37	0.19	0.06	0.99	0.12	0.12	**3.72**
		**2027**	2.26	0.49	0.16	0.07	1.20	0.16	0.16	**4.50**
	**South Korea**	**2022**	0.91	0.24	0.19	0.05	4.95	0.23	1.28	**7.85**
		**2027**	1.56	0.27	0.25	0.13	5.68	0.26	1.46	**9.60**
	**Taiwan**	**2022**	1.37	0.35	0.47	0.03	5.27	0.37	0.07	**7.93**
		**2027**	2.31	0.39	0.60	0.08	5.64	0.40	0.08	**9.49**
	**Thailand**	**2022**	1.68	0.56	0.24	0.29	7.91	0.54	0.98	**12.19**
		**2027**	1.85	0.67	0.31	0.42	10.03	0.67	1.51	**15.44**

CKD, chronic kidney disease; KRT, kidney replacement therapy.
[*]The annual national healthcare budget represents 100% for each table row.
[†]In settings without a single public healthcare system, the model was adapted to use a commercial or equivalent framework, or a mixed funding model. For example, in the case of the USA, costs were split into Medicare and commercial categories; for India, costs were split into four components: charitable, private, employment insurance and public. For these countries, distinct input data for each aspect of the funding models were used.

‡UAE expatriate population was modelled separately from the Emirati population owing to distinct population demographics requiring separate input data.

CC-BY 2024. Projecting the economic burden of chronic kidney disease at the patient level (Inside CKD): a microsimulation modelling study. S. Chadban *et al*. (Table S7) https://doi.org/10.1016/j.eclinm.2024.102615.[38] This work is licensed under a CC-BY licence http://creativecommons.org/licenses/by/4.0/).

Table S8. Projected cumulative transitions from CKD stage G3 to G4 and G4 to G5 (kidney failure) (2022–2027) per KDIGO uACR category in the diagnosed CKD population

Country/region		Transition from CKD stage G3 to G4, KDIGO uACR category A1	A2	A3	Transition from CKD stage G4 to G5 (kidney failure), KDIGO uACR category A1	A2	A3
Americas	Brazil, n (%)	88 371 (78.9)	7355 (6.6)	16 209 (14.5)	47 181 (87.8)	2251 (4.2)	4278 (8.0)
	Canada, n (%)	153 029 (83.8)	28 093 (15.4)	1481 (0.8)	37 728 (85.9)	5951 (13.6)	239 (0.5)
	Colombia, n (%)	58 840 (84.0)	10 474 (15.0)	692 (1.0)	17 910 (90.9)	1690 (8.6)	93 (0.5)
	Mexico, n (%)	34 587 (50.3)	14 111 (20.5)	20 095 (29.2)	15 847 (76.0)	2452 (11.8)	2551 (12.2)
	USA, n (%)	833 375 (70.9)	284 269 (24.2)	58 077 (4.9)	146 621 (75.5)	39 961 (20.6)	7698 (4.0)
Europe	Belgium, n (%)	53 896 (85.8)	8511 (13.6)	373 (0.6)	20 004 (88.0)	2641 (11.6)	98 (0.4)
	Denmark, n (%)	1794 (68.7)	774 (29.6)	44 (1.7)	1476 (85.8)	235 (13.7)	9 (0.5)
	France, n (%)	15 325 (64.3)	8125 (34.1)	394 (1.7)	12 257 (88.6)	1534 (11.1)	44 (0.3)
	Germany, n (%)	105 544 (75.8)	32 180 (23.1)	1439 (1.0)	20 954 (80.8)	4866 (18.8)	122 (0.5)
	Greece, n (%)	3031 (73.4)	1034 (25.0)	65 (1.6)	2549 (90.9)	244 (8.7)	12 (0.4)
	Hungary, n (%)	2392 (66.8)	1138 (31.8)	50 (1.4)	2031 (86.5)	303 (12.9)	15 (0.6)
	Italy, n (%)	11 685 (75.4)	3290 (21.2)	516 (3.3)	7023 (89.9)	677 (8.7)	111 (1.4)
	Netherlands, n (%)	4679 (64.4)	2448 (33.7)	143 (2.0)	3124 (86.4)	473 (13.1)	18 (0.5)
	Poland, n (%)	8380 (64.9)	3572 (27.7)	960 (7.4)	5733 (84.0)	904 (13.3)	185 (2.7)
	Romania, n (%)	3169 (75.2)	995 (23.6)	52 (1.2)	3427 (94.4)	189 (5.2)	14 (0.4)
	Spain, n (%)	143 848 (87.8)	18 782 (11.5)	1294 (0.8)	37 244 (90.7)	3651 (8.9)	189 (0.5)
	Sweden, n (%)	2068 (67.5)	823 (26.9)	173 (5.6)	1319 (86.2)	183 (12.0)	29 (1.9)
	Türkiye, n (%)	221 591 (73.3)	69 016 (22.8)	11 903 (3.9)	33 906 (75.7)	9440 (21.1)	1462 (3.3)
	UK, n (%)	276 353 (84.5)	48 357 (14.8)	2263 (0.7)	59 419 (85.5)	9650 (13.9)	408 (0.6)
Middle East	Israel, n (%)	12 545 (68.2)	4745 (25.8)	1103 (6.0)	4945 (72.7)	1579 (23.2)	281 (4.1)
	Saudi Arabia, n (%)	91 767 (79.3)	19 309 (16.7)	4586 (4.0)	45 116 (86.7)	5622 (10.8)	1275 (2.5)
	UAE, n (%)	249 (53.5)	170 (36.6)	46 (9.9)	385 (83.2)	65 (14.0)	13 (2.8)
Asia-Pacific	Australia, n (%)	30 908 (73.9)	9596 (22.9)	1337 (3.2)	9332 (80.2)	2059 (17.7)	242 (2.1)
	China, n (%)	586 467 (75.2)	168 680 (21.6)	24 396 (3.1)	138 874 (79.9)	31 248 (18.0)	3615 (2.1)
	India[†], n (%)	767 609 (82.9)	134 946 (14.6)	23 943 (2.6)	109 468 (85.2)	16 695 (13.0)	2258 (1.8)
	Japan, n (%)	1 080 736 (78.9)	261 365 (19.1)	28 267 (2.1)	123 393 (79.9)	28 234 (18.3)	2822 (1.8)
	Philippines, n (%)	11 564 (69.5)	1558 (9.4)	3523 (21.2)	9818 (88.5)	413 (3.7)	857 (7.7)
	Singapore, n (%)	1418 (49.4)	1182 (41.2)	268 (9.3)	1540 (76.9)	391 (19.5)	71 (3.5)
	South Korea, n (%)	79 540 (74.3)	23 902 (22.3)	3550 (3.3)	26 755 (85.0)	4177 (13.3)	559 (1.8)
	Taiwan, n (%)	35 799 (73.8)	11 126 (23.0)	1553 (3.2)	11 280 (83.4)	1984 (14.7)	258 (1.9)
	Thailand, n (%)	363 263 (82.0)	67 852 (15.3)	11 683 (2.6)	113 286 (85.8)	16 331 (12.4)	2404 (1.8)
	Total, n	**5 083 822**	**1 247 778**	**220 478**	**1 069 945**	**196 093**	**32 230**

	**Percentage unweighted average, % (SD)**	**72.8 (9.8)**	**22.2 (8.1)**	**5.0 (6.3)**	**84.4 (5.3)**	**13.2 (4.8)**	**2.4 (2.7)**
	**Percentage weighted average, %**	**77.6**	**19.0**	**3.4**	**82.4**	**15.1**	**2.5**

CKD, chronic kidney disease; KDIGO, Kidney Disease: Improving Global Outcomes; SD, standard deviation; uACR, urine albumin–creatinine ratio.

CC-BY 2024. Projecting the population level burden of CKD progression according to urine albumin-to-creatinine ratio categories. L. De Nicola *et al*. (Table S1) https://doi.org/10.1016/j.ekir.2024.09.021.[40] This work is licensed under a Creative Commons CC-BY-NC-ND licence http://creativecommons.org/licenses/by/4.0/).

Table S9. Projected cumulative incidence of cardiorenal complications and all-cause mortality (2022–2027) per KDIGO uACR category in the diagnosed CKD population (CKD stages G3–G5) for 31 countries and regions

Country/region		Heart failure			Myocardial infarction			Stroke			All-cause mortality		
		KDIGO uACR category			KDIGO uACR category			KDIGO uACR category			KDIGO uACR category		
		A1	A2	A3	A1	A2	A3	A1	A2	A3	A1	A2	A3
	**Brazil, n (%)**	596 406 (91.3)	20 030 (3.1)	36 772 (5.6)	311 198 (90.4)	11 932 (3.5)	21 057 (6.1)	257 825 (84.3)	14 863 (4.9)	33 189 (10.9)	4 056 180 (82.7)	233 188 (4.8)	614 907 (12.5)
	**Canada, n (%)**	195 046 (88.3)	24 677 (11.2)	1067 (0.5)	101 665 (88.0)	13 214 (11.4)	602 (0.5)	150 062 (83.3)	28 595 (15.9)	1500 (0.8)	938 265 (82.6)	185 171 (16.3)	12 042 (1.1)
**Americas**	**Colombia, n (%)**	123 058 (93.6)	7990 (6.1)	413 (0.3)	65 735 (93.5)	4390 (6.2)	209 (0.3)	14 374 (90.6)	1396 (8.8)	96 (0.6)	552 495 (88.7)	65 603 (10.5)	4757 (0.8)
	**Mexico, n (%)**	160 295 (76.4)	25 887 (12.3)	23 673 (11.3)	192 673 (76.4)	30 509 (12.1)	28 974 (11.5)	77 086 (64.3)	19 913 (16.6)	22 922 (19.1)	1 203 806 (59.7)	337 077 (16.7)	475 375 (23.6)
	**USA, n (%)**	1 120 440 (79.2)	249 829 (17.7)	43 954 (3.1)	395 807 (79.3)	86 535 (17.3)	16 619 (3.3)	912 004 (70.0)	323 086 (24.8)	67 423 (5.2)	5 246 667 (65.1)	2 206 843 (27.4)	601 431 (7.5)
	**Belgium, n (%)**	49 350 (89.1)	5849 (10.6)	218 (0.4)	28 491 (88.8)	3438 (10.7)	157 (0.5)	25 410 (84.6)	4443 (14.8)	189 (0.6)	377 644 (84.2)	67 467 (15.0)	3574 (0.8)
	**Denmark, n (%)**	27 644 (90.1)	2917 (9.5)	118 (0.4)	20 749 (89.9)	2232 (9.7)	91 (0.4)	23 225 (84.3)	4092 (14.9)	222 (0.8)	178 124 (83.0)	34 305 (16.0)	2136 (1.0)
	**France, n (%)**	127 274 (89.1)	14 989 (10.5)	645 (0.5)	38 884 (89.8)	4206 (9.7)	217 (0.5)	95 935 (83.9)	17 408 (15.2)	1066 (0.9)	988 901 (81.8)	206 987 (17.1)	12 870 (1.1)
	**Germany, n (%)**	26 506 (84.1)	4743 (15.1)	250 (0.8)	4869 (83.5)	943 (16.2)	22 (0.4)	10 928 (76.9)	3140 (22.1)	144 (1.0)	692 284 (76.3)	203 717 (22.5)	11 010 (1.2)
	**Greece, n (%)**	38 121 (93.9)	2383 (5.9)	94 (0.2)	53 569 (93.1)	3813 (6.6)	175 (0.3)	43 624 (90.2)	4507 (9.3)	253 (0.5)	322 503 (88.8)	38 182 (10.5)	2609 (0.7)
	**Hungary, n (%)**	79 801 (91.3)	7363 (8.4)	259 (0.3)	17 560 (90.6)	1766 (9.1)	59 (0.3)	36 369 (85.8)	5788 (13.7)	236 (0.6)	324 471 (84.5)	56 758 (14.8)	2940 (0.8)
	**Italy, n (%)**	105 128 (92.8)	7275 (6.4)	842 (0.7)	150 126 (92.1)	11 392 (7.0)	1481 (0.9)	120 477 (88.2)	14,017 (10.3)	2061 (1.5)	783 685 (87.6)	93 690 (10.5)	17 733 (2.0)
**Europe**	**Netherlands, n (%)**	59 182 (89.8)	6491 (9.8)	260 (0.4)	66 100 (89.8)	7205 (9.8)	339 (0.5)	47 633 (84.0)	8604 (15.2)	447 (0.8)	389 071 (82.9)	75 552 (16.1)	4599 (1.0)
	**Poland, n (%)**	162 053 (87.7)	19 029 (10.3)	3620 (2.0)	154 777 (88.0)	17 562 (10.0)	3527 (2.0)	313 354 (86.9)	40 278 (11.2)	6928 (1.9)	831 452 (74.9)	220 417 (19.9)	58 463 (5.3)
	**Romania, n (%)**	78 767 (96.8)	2509 (3.1)	92 (0.1)	71 150 (96.9)	2179 (3.0)	94 (0.1)	155 272 (96.6)	5256 (3.3)	205 (0.1)	303 490 (94.5)	16 852 (5.2)	793 (0.2)
	**Spain, n (%)**	194 883 (93.0)	14 066 (6.7)	705 (0.3)	101 151 (92.3)	8113 (7.4)	303 (0.3)	91 161 (88.1)	11 558 (11.2)	800 (0.8)	1 244 076 (88.8)	146 141 (10.4)	10 074 (0.7)
	**Sweden, n (%)**	41 047 (90.8)	3637 (8.0)	535 (1.2)	14 245 (90.7)	1255 (8.0)	199 (1.3)	25 ,071 (85.7)	3493 (11.9)	685 (2.3)	205 190 (84.4)	30 728 (12.6)	7055 (2.9)
	**Türkiye, n (%)**	121 290 (79.6)	26 731 (17.6)	4277 (2.8)	62 727 (78.1)	15 396 (19.2)	2162 (2.7)	53 298 (69.4)	19 942 (26.0)	3567 (4.6)	988 877 (69.4)	366 716 (25.7)	68 661 (4.8)
	**UK, n (%)**	174 043 (88.1)	22 517 (11.4)	988 (0.5)	102 225 (88.4)	12 746 (11.0)	639 (0.6)	241 497 (83.2)	46 239 (15.9)	2381 (0.8)	2 025 603 (83.1)	388 633 (15.9)	23 252 (1.0)

**Middle East**	**Israel, n (%)**	10 088 (75.2)	2849 (21.3)	469 (3.5)	4419 (75.9)	1171 (20.1)	233 (4.0)	6991 (64.2)	3154 (28.9)	751 (6.9)	101 743 (63.0)	47 428 (29.4)	12 270 (7.6)
	**Saudi Arabia, n (%)**	78 493 (84.4)	11 987 (12.9)	2543 (2.7)	42 459 (85.1)	6236 (12.5)	1188 (2.4)	21 650 (74.7)	5882 (20.3)	1436 (5.0)	221 803 (70.9)	71 320 (22.8)	19 516 (6.2)
	**UAE, n (%)**	4083 (84.7)	604 (12.5)	132 (2.7)	2250 (85.0)	326 (12.3)	72 (2.7)	1811 (76.8)	436 (18.5)	111 (4.7)	7757 (70.3)	2499 (22.7)	775 (7.0)
**Asia-Pacific**	**Australia, n (%)**	66 731 (85.3)	10 221 (13.1)	1281 (1.6)	17 191 (84.6)	2774 (13.6)	359 (1.8)	22 194 (74.7)	6557 (22.1)	950 (3.2)	230 469 (74.0)	68 840 (22.1)	12 161 (3.9)
	**China, n (%)**	257 419 (90.2)	23 600 (8.3)	4502 (1.6)	1 533 796 (84.9)	239 856 (13.3)	32 903 (1.8)	1 394 268 (79.6)	314 073 (17.9)	43 980 (2.5)	9 080 382 (77.1)	2 289 022 (19.4)	402 140 (3.4)
	**India†, n (%)**	1 229 418 (89.2)	128 180 (9.3)	20 107 (1.5)	1 274 460 (88.5)	145 013 (10.1)	21 144 (1.5)	965 288 (84.3)	154 183 (13.5)	25 144 (2.2)	8 434 339 (82.9)	1 444 771 (14.2)	290 007 (2.9)
	**Japan, n (%)**	813 771 (84.2)	138 653 (14.3)	14 535 (1.5)	886 547 (83.6)	157 323 (14.8)	16 391 (1.5)	518 357 (77.4)	134 388 (20.1)	16 923 (2.5)	4 300 349 (77.2)	1 111 792 (20.0)	156 997 (2.8)
	**Philippines, n (%)**	207 878 (91.5)	6816 (3.0)	12 401 (5.5)	222 908 (91.3)	7521 (3.1)	13 837 (5.7)	135 087 (85.5)	7012 (4.4)	15 892 (10.1)	1 003 425 (83.2)	55 991 (4.6)	147 347 (12.2)
	**Singapore, n (%)**	6070 (88.4)	637 (9.3)	162 (2.4)	20 239 (79.0)	4518 (17.6)	872 (3.4)	8653 (73.0)	2682 (22.6)	511 (4.3)	74 776 (69.3)	26 361 (24.4)	6775 (6.3)
	**South Korea, n (%)**	75 412 (84.0)	12 920 (14.4)	1476 (1.6)	21 071 (84.3)	3507 (14.0)	409 (1.6)	45 108 (77.5)	11 427 (19.6)	1655 (2.8)	322 801 (76.1)	86 939 (20.5)	14 301 (3.4)
	**Taiwan, n (%)**	35 163 (85.8)	5161 (12.6)	663 (1.6)	23 261 (84.7)	3725 (13.6)	464 (1.7)	12 868 (76.7)	3414 (20.3)	502 (3.0)	181 999 (76.6)	47 824 (20.1)	7815 (3.3)
	**Thailand, n (%)**	154 247 (91.8)	11 455 (6.8)	2360 (1.4)	532 826 (85.6)	77 882 (12.5)	12 068 (1.9)	218 130 (80.5)	45 674 (16.9)	7223 (2.7)	1 658 852 (77.3)	403 642 (18.8)	84 494 (3.9)
	**Total, n**	6 419 107	821 997	179 413	6 535 131	888 679	176 867	6 045 011	1 265 500	259 392	47 271 478	10 630 456	3 088 878
	**Percentage unweighted average, % (SD)**	87.7 (5.1)	10.4 (4.3)	1.9 (2.2)	86.8 (5.2)	11.1 (4.4)	2.0 (2.3)	80.8 (7.6)	15.8 (6.2)	3.3 (3.9)	78.7 (8.2)	17.0 (6.4)	4.3 (4.8)
	**Percentage weighted average, %**	86.5	11.1	2.4	86.0	11.7	2.3	79.9	16.7	3.4	77.5	17.4	5.1

KDIGO, Kidney Disease; Improving Global Outcomes; SD, standard deviation; uACR, urine albumin–creatinine ratio.

CC-BY 2024. Projecting the population level burden of CKD progression according to urine albumin-to-creatinine ratio categories. L. De Nicola *et al*. (Tables S2 and S3) https://doi.org/10.1016/j.ekir.2024.09.021.[40] This work is licensed under a Creative Commons CC-BY-NC-ND licence http://creativecommons.org/licenses/by/4.0/).

Table S10. Mean (range) diagnosis rates per 100 000 screened persons across 31 countries/regions

Outcome		General population ≥ 45 years old			General population ≥ 65 years old		
		Standard practice	2 eGFR only	2 eGFR and 1 uACR	Standard practice	2 eGFR only	2 eGFR and 1 uACR
Diagnosed via programme	RASi	N/A	3973 (1076–8715)	8111 (2151–14 799)	N/A	7774 (2430–15 872)	13 553 (3531–22 638)
	Untreated	N/A	3111 (882–8296)	5944 (2064–10 608)	N/A	5008 (853–9454)	8575 (1294–15 329)
Diagnosed via standard practice		459 (155–748)	391 (90–686)	337 (120–649)	629 (214–1056)	470 (166–811)	419 (108–736)
Screened, negative		N/A	64 878 (31 253–91 132)	57 961 (26 178–82 986)	N/A	57 773 (14 881–81 076)	48 478 (13 361–64 886)

CKD, chronic kidney disease; eGFR, estimated glomerular filtration rate; RASi, renin–angiotensin system inhibitor; SGLT2i, sodium–glucose co-transporter 2 inhibitor; uACR, urine albumin–creatinine ratio.

Note: the simulated cohort comprises only people without a CKD diagnosis who are eligible for screening, and not all patients are assumed to undergo screening. After enrolment of screening in year 1, these values will correspond to all incident patients subsequently diagnosed with CKD. Hence, the smaller values relating to current practice will reflect new diagnoses according to current pathways for diagnosis in each country/region and will not include the prevalent population diagnosed with CKD prior to the simulation. In line with KDIGO 2012 guidelines, *Inside CKD* assumed that only RASi therapy was used following diagnosis and that it was prescribed according to national eligibility criteria.[42] More recent guidelines (KDIGO 2024) recommend SGLT2i therapy alongside RASis for most patients with CKD.[6]

CC-BY 2025. Inside CKD: Cost-effectiveness of Multinational Screening for CKD. N Tangri *et al*. (Table 1) https://doi.org/10.1016/j.ekir.2025.01.020.[39] This work is licensed under a Creative Commons CC-BY-NC-ND licence http://creativecommons.org/licenses/by/4.0/).
